# The tropical-subtropical coupling in the Southeast Atlantic from the perspective of the northern Benguela upwelling system

**DOI:** 10.1371/journal.pone.0210083

**Published:** 2019-01-22

**Authors:** Lydia Siegfried, Martin Schmidt, Volker Mohrholz, Hans Pogrzeba, Pascal Nardini, Michael Böttinger, Gerik Scheuermann

**Affiliations:** 1 Leibniz Institute for Baltic Sea Research Warnemuende, Rostock, Germany; 2 Institute for Computer Science, Leipzig University, Leipzig, Germany; 3 Deutsches Klimarechenzentrum (DKRZ), Hamburg, Germany; Universidade de Aveiro, PORTUGAL

## Abstract

In the Benguela upwelling system, the environmental conditions are determined to a large extent by central water masses advected from remote areas onto the shelf. The origin, spreading pathways and fate of those water masses are investigated with a regional ocean model that is analysed using Eulerian passive tracers and on the basis of Lagrangian trajectories. Two major water masses influencing the Benguela upwelling system are identified: tropical South Atlantic Central Water (SACW) and subtropical Eastern South Atlantic Central Water (ESACW). The spreading of tropical waters into the subtropical Benguela upwelling system is mediated by equatorial currents and their continuation in the Southeast Atlantic. This tropical-subtropical connection has been attributed to signal propagation in the equatorial and coastal waveguides. However, there exists an additional spreading path for tropical central water in the open ocean. This mass transport fluctuates on a seasonal scale around an averaged meridional transport in Sverdrup balance. The inter-annual variability of the advection of tropical waters is related to Benguela Niños, as evidenced by the 2010/2011 event. The northern Benguela upwelling system is a transition zone between SACW and ESACW since they encounter each other at about 20°S. Both water masses have seasonal variable shares in the upwelled water there. To summarise the main pathways of central water mass transport, an enhanced scheme for the subsurface circulation in the Southeast Atlantic is presented.

## Introduction

The Benguela upwelling system belongs to the major eastern boundary systems, which are highly productive regions of the oceans. Its northern part is influenced by both tropical and subtropical central waters. Poleward propagating warm tropical South Atlantic Central Water (SACW) supplies nutrients but only low amounts of oxygen to the Benguela ecosystem in austral summer, whereas the eastern SACW (ESACW) from the “Cape Cauldron” [1] carries oxygen to the north in austral winter [2]. The southernmost extension of SACW is the Lüderitz upwelling cell [3], which marks the southern boundary of the northern Benguela upwelling system. Geographically, the southern boundary of the northern Benguela is the Lüderitz Orange River cone (LUCORC) barrier [4–6]. To the north, the Benguela upwelling system is bounded by the Angola-Benguela frontal zone (ABFZ), which is usually found north of the mouth of the Kunene river at 17°S during austral winter, and south of the Kunene mouth during austral summer [7–10].

The seasonally varying poleward propagation of warm tropical water determines the seasonal cycle of the ocean temperature in the northern Benguela. Changing temperature is the most obvious variability, but the same way salinity, oxygen and nutrient concentration or the drift of fish larvae depend on the alternating influence of SACW and ESACW [2, 11]. Both water masses, SACW and ESACW cover a different range of potential temperature and salinity. Remarkably, a water mass analysis revealing the varying proportion of the two water masses explains the observed oxygen and nutrient variability to a large extent [2, 11–13]. Local processes may be important, but are able only to moderate the fluctuations imposed by the varying influence of different central water masses [14]. The seasonal cycle of the water mass distribution in the northern Benguela may have strong anomalies. During extreme warm events, known as Benguela Niños [15], above-average sea surface temperatures are observed along the coast of Angola and Namibia. The major warm water intrusions of 1995 [16] and 2010/11 [17] resulted in ecosystem regime shifts of both the pelagic [18] and benthic communities [19]. Benguela Niños are understood as events of intensified tropical-subtropical coupling with exceptionally high poleward transport of SACW into the northern Benguela upwelling system [20].

The properties of central water masses are formed in their source region but can be modified along their pathway, for example, by diapycnal diffusion of heat and salt or by oxygen consumption. Knowledge on both the characteristics of the source region and on the pathway towards the Benguela upwelling system helps to understand the variability of its hydrographic conditions in more detail. Whereas time series of the hydrographic variability in the northern Benguela are well studied, the pathways of the spreading of tropical water into the subtropical Benguela have not yet been conclusively clarified. This motivates the analysis presented in this paper, which reveals not only more details on the pathways but also on ocean time scales emerging from the water mass exchange between the tropical and the subtropical Atlantic.

**Fig 1:**
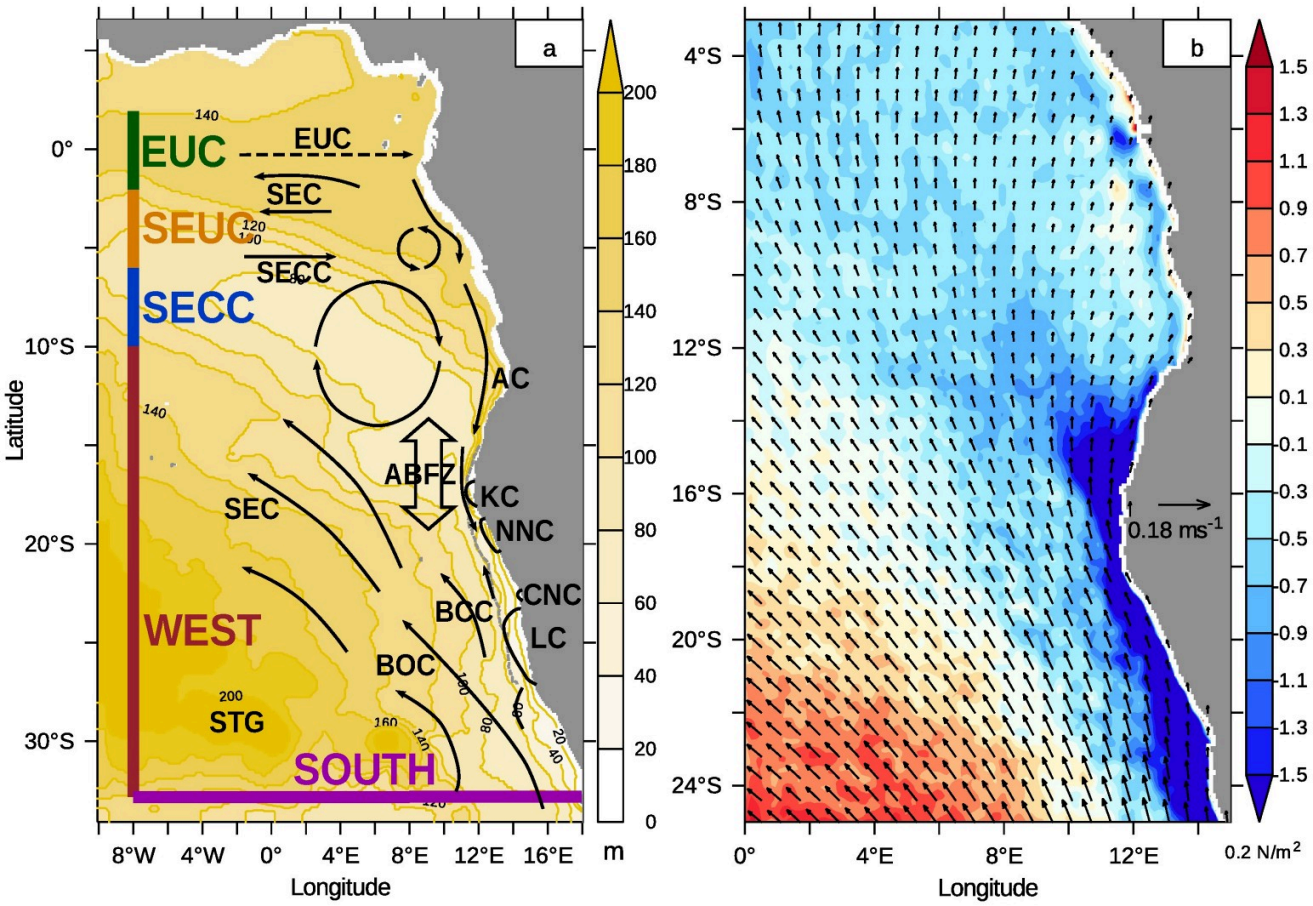
Model domain, depth of *σ*_0_ = 26.2 and wind stress. (a) The release regions of the passive tracers are plotted as coloured lines parallel to the model boundaries (cf. Table 1). The surface and near-surface currents and the upwelling cells of the northern Benguela are redrawn from [21]. Please refer to the text for abbreviations. The depth [m] of the *σ*_0_ = 26.2-isopycnal which separates surface from central water [22] is shaded in yellow. The potential density was calculated from modelled temperature and salinity. (b) Wind stress [N m^−2^] is represented by vectors and wind stress curl [10^−7^ N m^−3^] is colour coded. Areas of negative wind stress curl are shaded in blue. Shown are model results from January of the monthly climatology from 2002 to 2016.

Fig 1 gives an overview of the typical wind fields, surface and subsurface currents and upwelling cells. The surface and subsurface currents and the upwelling cells of the northern Benguela are redrawn after Hardman-Mountford et al. 2003 [21]. Fig 1 also comprises the domain of the numerical circulation model used during this work. The relatively persistent wind stress pattern belongs to the Southeast Trades (cf. Fig 1b), which are in balance with the pressure difference between a semi-permanent high-pressure system, the South Atlantic High (also known as St. Helena High), and thermal lows which develop over Africa [23].

We start the overview of the current system with the surface circulation south of the ABFZ which is governed by the Benguela Current and the South Equatorial Current (SEC) forming the southwestern boundary of the Benguela upwelling system. The Benguela Current branches at approximately 26°S into an oceanic (BOC) and a coastal (BCC) current. The BOC and the SEC form the eastern limb of the basin-wide cyclonic Subtropical Gyre (STG) [24]. It carries subtropical ESACW northward that emerges from the mixing of SACW and Indian Central Water [25]. ESACW is also found periodically in the northern Benguela especially during the upwelling season. The SEC does not undergo any subduction within the considered area and leaves the model domain as a surface current.

The part of the current system contributing to the SACW circulation are the equatorial currents [26]. We do not consider the westward directed equatorial surface currents, which are confined to the sea surface and do not contribute to the tropical-subtropical coupling within the model domain. Below the surface currents, three branches of the equatorial current system, the Equatorial UnderCurrent (EUC), the South Equatorial UnderCurrent SEUC (missing in [21]) and the South Equatorial Counter Current (SECC) carry tropical SACW eastward. These currents bend poleward near the coast and feed into the Angola Current (AC). Warm SACW proceeds poleward along the African coast until it encounters cooler upwelled water in the area of the ABFZ. The ABFZ with its sharp gradients in sea surface temperature forms the northern boundary of the Benguela upwelling area. Here, the poleward surface flow detaches from the coast and bends off-shore, but the subsurface flow intermittently continues poleward as an undercurrent (PUC). Thus, tropical SACW can penetrate the northern Benguela system [10, 12, 27, 28].

The closed circle in Fig 1 symbolises the Angola Gyre [23, 29, 30] encircling the Angola Dome [31, 32]. This area is harbouring an extended oxygen minimum zone [33, 34] and is often described as a shadow zone with reduced water exchange with the ambient ocean. However, the heat budget in the area of the Angolan Dome might be considerably influenced by Rossby waves that radiate from the African coast [35].

The poleward transport of SACW is mostly attributed to Kelvin- and shelf-wave dynamics in the equatorial and coastal waveguide. Equatorial Kelvin waves can be excited by fluctuating winds in the western tropical Atlantic. Propagating eastward, the waves modulate the strength of the equatorial currents. They are deflected at the coast to continue poleward as coastal Kelvin waves [36–38]. Signals of such waves can be seen in altimeter-based sea surface height anomaly [39], but also in more detailed analysis in combination with a general circulation model [40–42]. The phase speed of these waves is estimated to be in the range of 0.8 ms^−1^ to 1.1 ms^−1^ [42] and 1.5 ms^−1^ to 2.1 ms^−1^ [41]. However, coastally trapped waves can also be forced locally. Richter et al. [43] found a significant contribution of local meridional wind anomalies to the development of Benguela Niños.

The volume budget analysis for the northern Benguela in Fig 19 of Lass et al. 2000 [44] supports a scenario, where the major SACW transport does not follow the coastal waveguide but joins the poleward current near the ABFZ. Hence, we query that the transport pathway of SACW only follows the equatorial and coastal waveguide. The wave signals visible in altimeter and SST data, indeed suggest such a propagation of tropical water. However, water transport takes place with currents. A wave signal may propagate through the equatorial and coastal waveguide to finally induce a poleward intrusion of warm SACW into the northern Benguela. This does neither mean that this warm water itself comes from the equator nor that it reached the northern Benguela following the coastal waveguide. On the contrary, the SACW may come from somewhere else and may be advected on a different path to the northern part of the ABFZ, where it can be pushed finally into the northern Benguela by currents related to a wave pulse.

Following the analysis of Lass et al., 2008 [24], we assume the first baroclinic mode of westward propagating Rossby waves as the major mechanism exporting poleward momentum from the coastal waveguide into the open ocean. This explains observed bands of subsurface currents away from the coastal waveguide and leads to the question if a Sverdrup balance between the vorticity flux from the wind stress curl and the meridional transport of planetary vorticity develops [45, 46]. This adjustment of flow to the wind stress pattern is mediated by Rossby waves radiating from the eastern ocean boundary [47]. Since it takes up to an year until the first baroclinic mode crosses the area of interest [24] and the wind pattern undergoes a significant seasonal cycle, this adjustment will never be complete. Instead we expect the poleward flow to fluctuate around an average flow pattern in Sverdrup balance with wind stress curl.

The last element of this circulation scheme is upwelling. We reference the term upwelling for a combination of all turbulent processes that bring water to the surface and wind-driven upwelling which manifests itself in upward directed vertical velocities, here in detail wind-driven coastal upwelling and wind stress curl driven upwelling [13, 48]. The poleward spreading of tropical water with the PUC in combination with upwelling extends the influence of tropical water into the subtropics [49, 50].

Wind stress and wind stress curl determine upwelling, but the three-dimensional circulation patterns in the Benguela upwelling system can only be understood in the context of the coastal jet, coastally trapped waves and the PUC [48, 51]. A structured coastline and varying shelf width arrange these patterns [52] to localised so-called upwelling cells [53, 54]. Their position and approximate extent is shown in Fig 1. The most prominent northern Benguela upwelling cells are located at 18°S (KC—Kunene Cell), 20°S (NNC—Northern Namibian Cell) and at 23°S (CNC—Central Namibian Cell). The Lüderitz Cell (LC, 27°S) as the largest upwelling cell in the Benguela upwelling area [53], is driven by the perennial upwelling-favourable winds. It acts like a natural barrier between the northern and southern Benguela upwelling system [6]. The poleward flow of tropical water with the PUC is terminated stepwise within the northern Benguela upwelling system [44] and does not continue through this cell.

As demonstrated with the drifter experiment in [24], the surface flow bends northward in the ABFZ and becomes part of the Benguela Current. Also the subsurface flow merges into the Benguela Current after upwelling within the different upwelling cells in the northern Benguela. This motivates a more detailed analysis of the hydrographic variability within the northern Benguela. Results from a realistic numerical circulation model suggest some fundamental modification and refinement of the circulation scheme sketched in Fig 1.

Both the so called upwelling depth and the origin of the upwelling source water mass are crucial for the nutrient supply to the euphotic zone, but also for the atmosphere-ocean gas fluxes [14, 55].

First estimates of upwelling depth in the northern Benguela upwelling system ranged between 200m and 300m [56]. Subsequent temperature and salinity measurements [57–59] and references in Shannon (1989, [60]) confirmed this result. In contrast, an upwelling depth of 50m to 100m was reported at Walvis Bay, 23°S [61], whereas the upwelled water south of 30°S originates from depths between 300m and 500m [62].

Since direct field measurements of currents are sparse, the study is based on numerical model results. We investigate the currents, but also analyse the long-distance connection between the tropical and subtropical Atlantic. The spreading of water masses is monitored over time scales from years to a decade. We go beyond the consideration of potential temperature and salinity to study water mass spreading and employ two complementary numerical methods: Lagrangian trajectories and Eulerian passive tracers.

Lagrangian trajectories [63–67] were used to examine the interchange of Indian and Atlantic waters [68, 69], the deep convection of North Atlantic Deep Water [70], or the extratropical-tropical exchange in the Western Atlantic [71]. Only the work of Nardini et al. in 2017 [67] has so far dealt with the tropical-subtropical coupling in the Southeast Atlantic. Their Lagrangian trajectory analysis is continued here with an analysis of upwelling trajectories. In addition, we use passive tracers [13, 72, 73] which follow the same advection-diffusion equation as potential temperature and salinity (with exception of a specific source term) and do not alter ocean dynamics. Such passive tracers have already been successfully used to track the origin of upwelled water masses in the Californian upwelling system [74]. Unlike the Lagrangian particles used by [67], the passive tracers are strictly conserved.

We will use both methods to answer the following questions:

- Which routes does the tropical SACW take to reach the northern edge of the Benguela upwelling area?
- Which physical processes contribute to the water transport along these routes?
- How are the central water masses transported within the northern Benguela?
- Is the water in the mixed layer solely composed of tropical SACW and subtropical ESACW?

Finally, we will discuss specific water mass spreading during the Benguela Niño of 2010/11.

## Materials and methods

### Modular Ocean Model—MOM

For the numerical simulations, the Modular Ocean Model version 5 (MOM) [75] coupled with the biogeochemical component model ERGOM [14, 76] is used. The model is configured as a regional model and covers the area from 10°W to 18°E and 8°N to 34°S. The coordinate system orients upward-directed velocities, i.e. upwelling, as positive and downwelling as negative. We use z*-coordinates [77]. This reduces numerical mixing by distributing the variations of the sea surface height over several model layers and allows for a high vertical model resolution on the shelf, starting with about 3m in the upper part and coarsening slowly below 120m depth [14]. The horizontal resolution varies from 8km on the Namibian shelf to 15km at the model boundaries. The total number of grid cells is 273 by 382 by 89, zonally, meridionally, and vertically, respectively. The baroclinic and barotropic time step amounts to 1200 s and 12 s, respectively. The simulation time covers the period from July 1999 to February 2017. Results were stored as 5 d-averages, a monthly climatology was calculated over the years 2002 to 2016 for temperature, salinity, currents and wind stress. The potential density is calculated by means of the TEOS-10 equation of state [78] from modelled temperature and salinity. The depth of the 26.2-isopycnal which separates surface water from central water [22] is calculated from the monthly climatology and shown in the left panel of Fig 1.

At the open model boundaries a radiation condition applies [72]. To account for processes outside the model domain, temperature, salinity and sea level are relaxed towards data derived from the ECCO2 model (CUBE92, [79]). Sea level data are prescribed daily, temperature and salinity data are updated with a three day period. Passive tracer concentrations are nudged towards zero at the boundary. All boundary data are linearly interpolated to the ocean time step.

Vertical mixing is determined using the K-profile parameterisation [80, 81]. The scheme delivers both the mixed layer depth and the surface boundary layer depth. Mixing is enhanced in the ocean interior over sloping topography. A positive definite advection scheme [82] is applied for all model tracers.

The ocean model is run as a forced model. Surface momentum, heat and fresh water fluxes are derived from the ERA-interim forecast data [83] using the Beljaars et al. (1995) bulk formulae [84]. Since the model performance depends on the accuracy of coastal winds and wind stress curl in the model forcing [48], the wind field on the coarse ERA-interim grid is replaced by scatterometer wind data with higher resolution. Compared with scatterometer based wind data, the ERA-interim forecast underestimates the meridional coastal wind speed, whereas open ocean wind speed is comparable with scatterometer winds. Consequently, the modelled coastal jet is too slow, but the off-shore increase of the meridional wind is larger in the ERA-interim data. The wind stress curl is related to a poleward transport of tropical water [45, 46]. Hence, scatterometer winds tend to drive less poleward transport of tropical water to the northern Benguela system. To use scatterometer data for modelling, gaps near the coast are filled by averaging over the nearest valid values of neighbouring grid points. QuikSCAT and ASCAT data are available as daily composites from three day data. To cover the strong daily wind variability, high pass filtered wind data from the ERA-interim forecasts are added so that the daily averages of these synthetic winds resemble the scatterometer derived daily winds. The quality of our model results is further limited by the discontinuity between the QuikScat and ASCAT missions whereby the forcing wind data are not a consistent data set [85].

Sensible and latent heat flux are calculated from sea surface temperature, the atmosphere temperature at 2m, and specific humidity. Incoming and outgoing longwave radiation is computed with a modified Stefan-Boltzman law. This way, the ocean model inherits skills and shortcomings of the prescribed atmospheric data set. In the northern Benguela, the ERA-interim solar radiation exceeds ship borne radiation data by about 100W/m^2^ [86]. Hence, solar radiation is recalculated from the total cloud cover using the Bodin radiation model [87]. The chlorophyll concentration of the ecosystem model [14] is used to evaluate the penetration of shortwave radiation into the ocean [88].

For model validation we compare the modelled SST with Reynolds daily SST, https://www.esrl.noaa.gov/psd/ (S1 Fig). The SST zonally averaged over a coastal strip of 1° width has a prominent seasonal cycle mostly due to the meridional migration of the ABFZ. The black line in S1 Fig shows the meridional position of the 20°C-isotherm from the Reynolds daily SST as oscillating around 16°S. It has a seasonal cycle and is found displaced equatorward from July to October and shifted poleward otherwise. There is a strong inter-annual variability of this motion. The Benguela Niño 2011 is seen as an exceptionally long-lasting poleward shift of this isotherm, but there are also a few years where the 20°C-isotherm extends far northward. The modelled SST (red line) shows a similar variability. Since the modelled SST has a general offset of 1.5° against the Reynolds SST, we show the 21°C-isotherm. The seasonal and inter-annual variability is consistent with the Reynolds SST, the amplitude of the displacement is smaller in the model results. To demonstrate the sensitivity of these results to details of the wind stress pattern, the blue line shows results from a model driven with ERA-interim winds. From the analytical theory of Fennel et al., 2012 [48] and the analysis of Junker et al., 2015 [45] we conclude that this larger model bias results from the underestimated coastal wind. The bias causes reduced coastal upwelling and reduced equatorward coastal jets in combination with enhanced curl-driven poleward transport of tropical water. This motivates the usage of scatterometer based wind data as model forcing, which allows for more realistic results. For details on model validation with respect to the cross-shelf distribution of temperature and salinity we refer to Mohrholz et al. 2014 [13]. Since the SST variability is dominated by the combined action of upwelling and flow convergence of the coastal branch of the Benguela Current and the Angola Current [89], we expect also a realistic variability of the tracer distribution.

### Sverdrup transport

The meridional component of the Sverdrup transport *M*_*y*,Sv_ is calculated from wind stress *τ*, the meridional derivative of the Coriolis parameter $\beta =\frac{\partial f}{\partial y}$ and density *ρ* by means of Eq 1 [90].

$$\begin{array}{c} M_{y,\text{Sv}}=\frac{{\left(\vec{\nabla }\times \vec{\tau }\right)}_{z}}{\beta \rho } \end{array}$$ (1)

This transport includes the Ekman balanced flow in the surface and the bottom boundary layer and a geostrophically balanced flow in the ocean interior, whose horizontal divergence at the sea surface and the sea floor balances the vertical velocity from Ekman pumping. Hence, assuming week stratification and low friction in the interior ocean, a stream function can be calculated by zonal integration of *M*_*y*,Sv_. The meridional derivative of the stream function also gives the zonal component of the Sverdrup balanced flow, *M*_*x*,Sv_.

The transport derived from the model results by vertical integration includes wind driven, but not Ekman balanced flow, i.e. coastal jets in the northern Benguela, and the thermohaline component of the meridional overturning circulation. To see solely the spreading of the central water masses, we exclude the flow within the Antarctic Intermediate Water and the North Atlantic Deep Water from consideration by limiting the vertical range of integration,

$$\begin{array}{cc} \vec{M} & =\int_{-500\;\text{m}}^{\eta }\vec{u}\text{d}z. \end{array}$$ (2)

This approach is supported by the reasoning of Small et al., 2015 [46] and references therein [91, 92]. They found only minor changes in the meridional transport at the eastern boundaries when choosing integration depths greater than 500 m.

### Passive tracers

#### Advection-diffusion equation

Unlike mere snapshots of the flow, passive tracers provide information about the time course of water spreading. Passive tracers obey the same advection-diffusion equation like other model tracers as heat content, salt-, oxygen- or nutrient concentration. The tracers have specific sources in limited regions *R* = *R*(*x*, *y*, *z*) bounded by a surface. In these regions, the tracer concentration is set to a constant value *c*_0_ = 1 m^−3^. This way the strength of the tracer source is proportionally to the flow through the tracer release region. The tracer concentration *c* is described by Eq 3

$$\begin{array}{c} \frac{\partial c}{\partial t}=[1-\theta (R)]\nabla \cdot ({\vec{F}}^{\text{adv}}+{\vec{F}}^{\text{dif}}) \end{array}$$ (3)

$$\begin{array}{c} c(t\geq t_{i},R)=c_{0},\;c\left(t\leq t_{i}\right)=0 \end{array}$$ (4)

where *θ* is the Heaviside step function equal to one within the source region and zero otherwise, ${\vec{F}}^{\text{adv}}$ and ${\vec{F}}^{\text{dif}}$ are advective and diffusive tracer fluxes. Note that the tracer start time, *t*_*i*_, is different from the model initialisation time. The tracer concentration within a Lagrangian fluid parcel has its maximum value within the release region and is decreasing by diffusive fluxes.

#### Release regions of passive tracers

**Fig 2:**
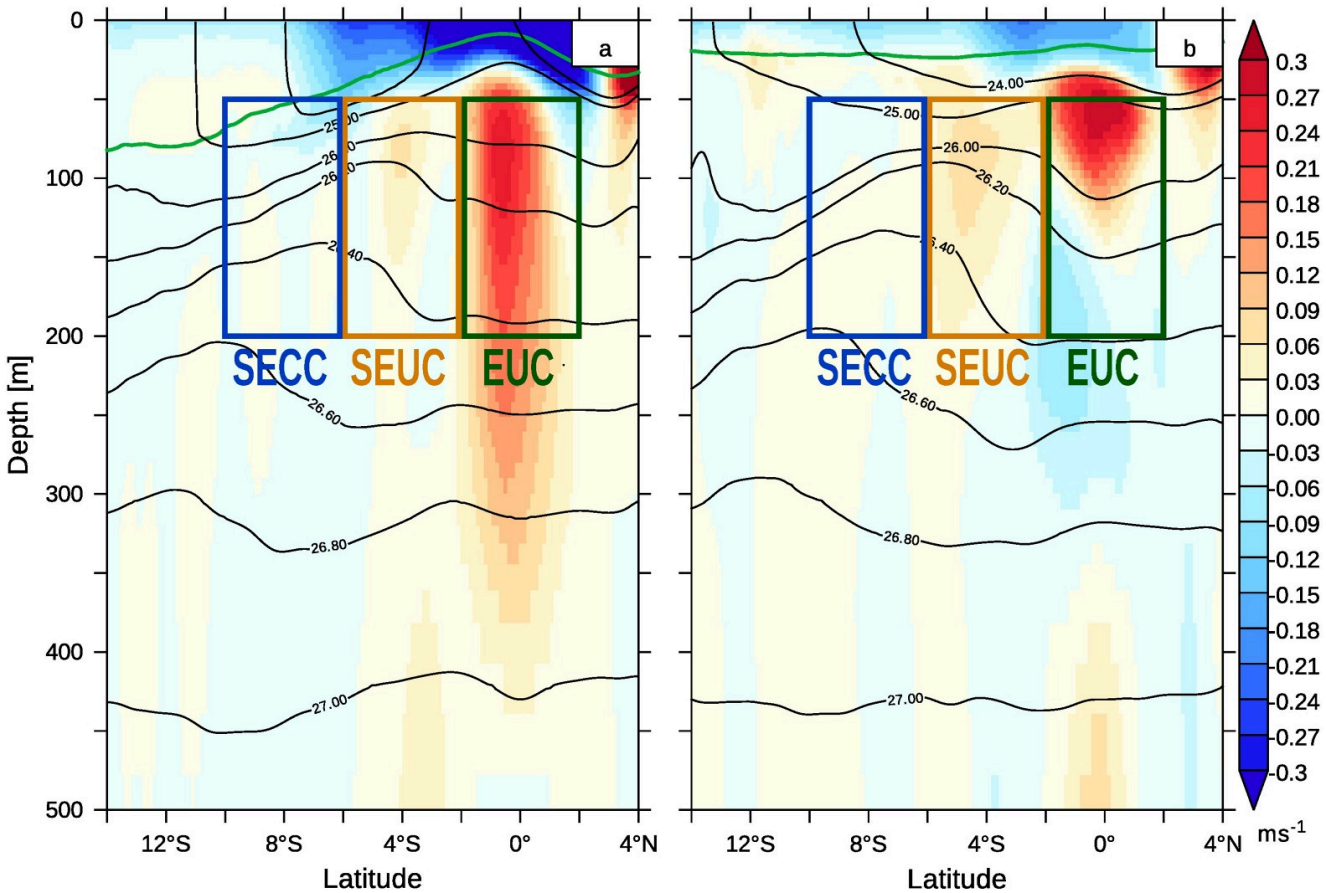
Zonal currents at 8°W and release regions of tropical passive tracers. Positive zonal velocities, i.e. eastward, are shaded in red. The release regions *R* of the EUC, SEUC and SECC tracers are shown (cf. Table 1). The mixed layer depth is plotted as a green line, black lines denote potential density. January (a) and July (b) of the monthly climatology from 2002 to 2016.

The release regions *R* of selected tracers are shown in the left panel of Fig 1 and are captured by Table 1. The last two columns specify the density range within the release regions. Two tracers are released on horizontal planes at depths of 200m and 550m within the entire model area to determine the relevant depth range for transport of upwelling water and the origin of the mixed layer water in the Southeast Atlantic. Another set of eight tracers is released on vertical sections parallel to the western and southern open model boundaries within a layer of 50m to 200m depth. Hence, four passive tracers are defined on a meridional section at 8°W from 2°N to 33°S. The section through the horizontal currents at 8°E, Fig 2, illustrates how the release regions of the passive tracers are embedded in the equatorial current system. The first region from 2°N and 2°S partly covers the core of the Equatorial UnderCurrent (EUC). The adjacent release region of the second tracer extends to 6°S and can be roughly assigned to the South Equatorial Undercurrent (SEUC). Part of the South Equatorial Counter Current (SECC) is represented by a third passive tracer released between 6°S and 10°S. Hereafter, these tracers are referred to as EUC, SEUC and SECC tracers and represent the spread of tropical SACW in the Southeast Atlantic. The release region of the fourth passive tracer south of 10°S was selected to be relatively large, since the flow in this area is directed outwards of the model domain. Note the different potential density *σ* in the release regions.

**Table 1:** Passive tracers: Release region and density range of their release region.

Name	Longitude		Latitude		Depth / m		Density range	
					min	max	min	max
EUC	8°W		2°S	2°N	50	200	22.807	26.525
SEUC	8°W		6°S	2°S	50	200	22.852	26.57
SECC	8°W		10°S	6°S	50	200	22.901	26.677
WEST	8°W		33°S	10°S	50	200	23.864	26.67
SOUTH 1	16°E	18°E	33°S		50	200	25.174	26.93
SOUTH 2	12°E	16°E	33°S		50	200	24.986	26.706
SOUTH 3	8°E	12°E	33°S		50	200	24.977	26.648
SOUTH 4	8°W	8°E	33°S		50	200	24.585	26.552
T300	10°W	18°E	34°S	8°N	300		25.362	27.013
T550	0°	18°E	30°S	8°N	550		26.465	27.199

The density intervals denote minimum and maximum of potential density *σ*_0_ = (*ρ* − 1000) / (kg m^−3^) within the tracer release regions.

To cover subtropical ESACW, that flows from the southern boundary into the model area with the Benguela Current, three tracers are released on a zonal section at 33°S. They are released in the same depth range as the western model boundary tracers (cf. Table 1). In the discussion below, SOUTH 1 to SOUTH 3 are summarised as “BC tracer”. A fourth tracer is released between 8°W and 8°E but does reach the northern Benguela upwelling system only with minor concentrations. Henceforth, we restrict our analysis to the BC tracer.

#### Spreading of tropical tracers

The spreading of passive tracers depends on the conservation and modification of the potential density. As long as diffusive heat and salt fluxes are small, the potential density of a moving water mass is conserved. The average tracer concentration within the density range of the tracer release region (Eq 5, Table 1, *σ*_min_ to *σ*_max_) characterises the relative amount of water originating from the tracer release region.

Moreover, when water enters the model domain, it traverses exactly one source region, Fig 1. After the equilibrium of the tracer distributions has established, this allows to check if the set of the source water masses is complete for a region in the model interior, for instance the northern Benguela upwelling system.

$$\begin{array}{c} \bar{c}\left(x,y,t\right)=\frac{\int_{z(\sigma_{\max})}^{z(\sigma_{\min})}c\;\text{d}z}{\int_{z(\sigma_{\max})}^{z(\sigma_{\min})}\;\text{d}z} \end{array}$$ (5)

Ocean salinity fluctuates around some equilibrium, which is established by transport processes between the different ocean areas. The model experiments with passive tracers show how such an equilibrium is formed. The observed time scales provide an estimate of the connectivity between remote ocean areas.

The resulting modelled passive tracer concentrations are not characterised by sharp tracer fronts which indicates that physical and numerical mixing are not small. Physical mixing is preferably isopycnal. Numerical mixing from limited model resolution takes place mostly along streamlines. The isopycnal component of both leads to a continuous distribution of tracer concentrations within a water mass from the same release region. Diapycnal mixing was assessed from the tracer concentrations in waters with potential density different from that in the tracer release region. The results do not reveal a considerable tracer transport outside the propagation paths presented in this publication.

In upwelling areas, water comes into contact with surface heat and fresh water fluxes and therefore potential density is no longer conserved. The formation of frontal structures and filaments enhances the surface between different water masses. Due to strong mixing, the central water masses loose their individual characteristics and a water mass analysis based on temperature and salinity becomes meaningless. Since the passive model tracers are strictly conserved, a quantification of the amount of source water masses in the newly formed water bodies is still possible.

#### Tracers in upwelling cells

In order to assess the temporal variability of the tracer amount on the shelf, the tracer concentration is averaged over a zonal section through the centre of the Kunene Cell at 18°S according to Eq 6 and similarly for the centre of the Lüderitz Cell at 27°S.

$$\begin{array}{c} c_{\text{KC}}\left(t\right)=\frac{\overset{\text{coast}}{\underset{200\;\text{m-isobath}}{\;\int \int \;}}c\left(x,y=18{}^{\circ}\text{S},z,t\right)\;\text{d}x\;\text{d}z}{\overset{\text{coast}}{\underset{200\;\text{m-isobath}}{\;\int \int \;}}\;\text{d}x\;\text{d}z} \end{array}$$ (6)

The averaging was carried out from the coast to the 200m isobath. Given a choice of 300m or 400m water depth, the results change quantitatively but the seasonal cycle remains unchanged. Since the shoreline of the Kunene Cell is almost meridionally aligned, the zonal section used in Eq 6 represents the cross-shore section across the shelf. Finally, the average is smoothed with a running mean of 30 d.

### Lagrangian trajectories

To assess connectivity between different ocean areas in more detail, we compute a space and time filling set of Lagrangian trajectories from the 5 d-averages of the three-dimensional velocity field for the entire model domain.

For the computation of the initial trajectory set, we seed one weightless particle at time *t*_*i*_ in each of the model grid cells and use a 4^th^-order Runge-Kutta scheme to compute particle pathlines for all remaining time steps. Whenever a grid cell is free of a particle after one advection time step, a new particle is seeded therein to guarantee a space filling set of trajectories covering the complete simulation time. The trajectories of these additional particles are also integrated backwards in time to obtain a dense and consistent representation of the vector field. Thus, the flow within each cell is always represented by at least 1 trajectory. Conservation laws are not valid for these particles since the total number of particles in the model domain is not constant.

We use a step size of 3 h for the computation of the trajectories. With these settings, the method yields on average 44 × 10^6^ particles, which is a spatially dense representation of the 3D flow in the 10 × 10^6^ model grid cells.

#### Upwelling particles

To characterise upwelling and mixing in the northern Benguela in more detail, a subset of trajectories is selected by using “predicates” that fulfil predefined conditions. This *pathline predicates* methodology was developed by Salzbrunn et al. and Born et al. [93–96] and first applied to ocean model data by Nardini et al. (2017, [67]). Nardini et al. classified their trajectories using a fixed depth and a predefined angle between the trajectories and the vertical axis. In this work, however, the trajectories were filtered by means of the mixed layer depth. We define “upwelling particles” through the following condition: particles from below of the mixed layer must traverse the mixed layer upwards. We consider only particles upwelling between 15°S and 27°S and between the coastline and the 400 m-isobath. The region covered by this definition is shown in Fig 1. To analyse spatio-temporal features of the combined effect of upward directed advection and mixing, the upwelling time, i.e. the time when the above criteria are met, is stored for each particle. This yields a time series of the number of upwelling particles. It should be noted that the trajectories are calculated solely from advection, but this “mixed layer criterion” takes also diffusive processes into consideration.

The contributions of different water masses to the mixed layer in the northern Benguela are quantified using the number of trajectories as a measure for water transport. Upwelling trajectories are named after the release regions already defined for the passive tracers (Table 1). Like passive tracers, these particles and their trajectories are referred to as EUC, SEUC etc. particles or trajectories. Unlike passive tracers, each particle classified this way is definitely brought to the surface in the northern Benguela later. All cells that include parts of the tracer release region are considered as tracer starting cells. All trajectories through these cells are tested for intersections with a surface of tracer release cells. In the rare case of intersections with several release regions, the one closest to the northern Benguela is used for naming. The EUC upwelling trajectories are further differentiated into “coastal” and “oceanic” trajectories. If such a trajectory intersects the zonal section at 6°S between 10°W and 8°E, it is assigned to be “oceanic”, otherwise to be “coastal”.

## Results and discussion

### Tropical-subtropical coupling

**Fig 3:**
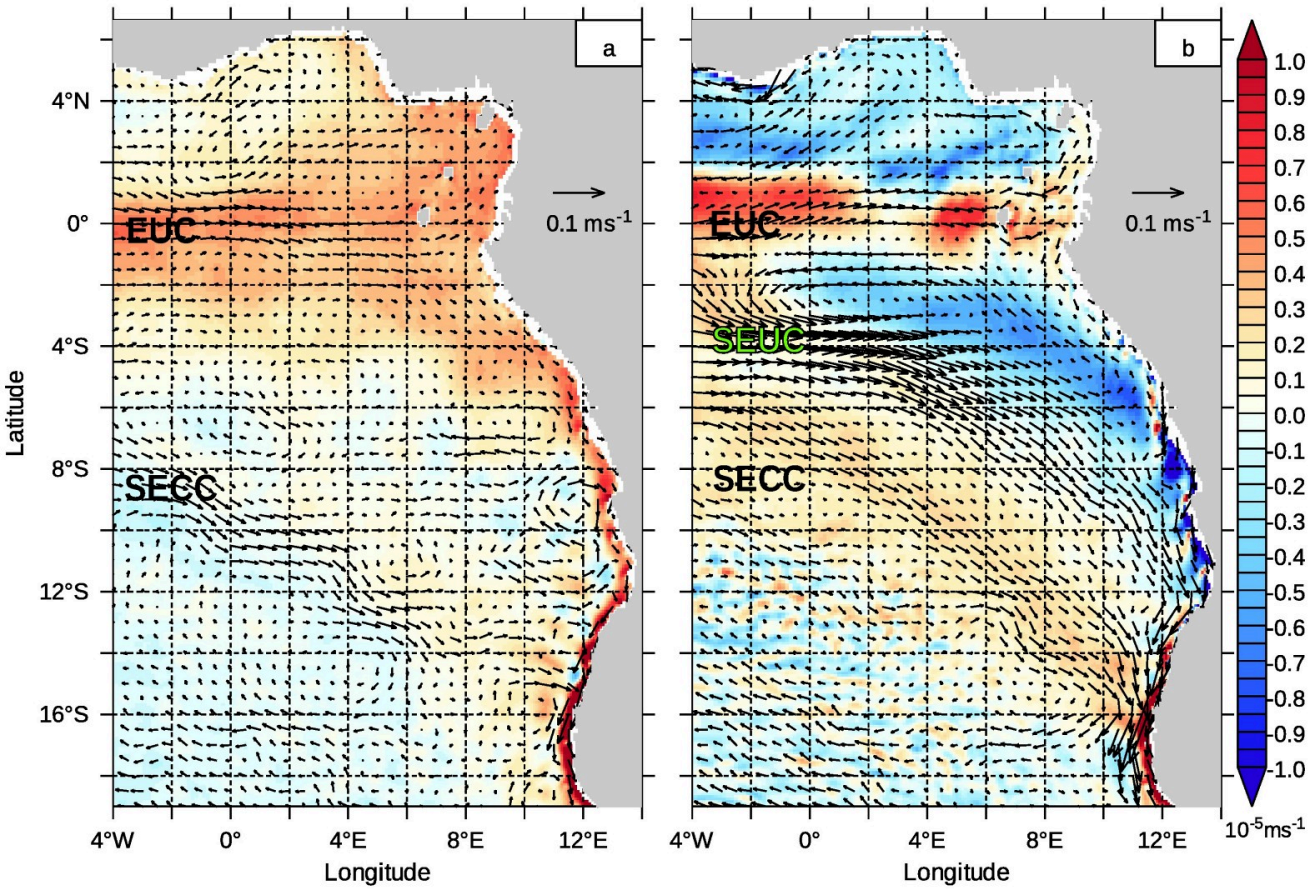
Currents in the subsurface layer. Horizontal currents averaged from 50m to 200m depth from a monthly climatology from 2002 to 2016. Colours represent the vertical velocity at 50m depth. Positive vertical velocity (red) is directed upward. (a): April (b) October.

The path of the central water masses to the Benguela upwelling system is part of the tropical-subtropical coupling in the Southeast Atlantic and will be discussed in the following. We will also analyse central waters spreading within the northern Benguela. The streams with enhanced horizontal velocity are identified as the Equatorial UnderCurrent (EUC), the South Equatorial Under Current (SEUC) and South Equatorial Counter Current (SECC). The designation of currents’ names was related to field measurements, which are mostly restricted to the area west of 10°W and north of 6°S or to the surface [97–102]. Due to the high temporal and spatial variability of these currents, the identification is sometimes ambiguous. The EUC is clearly identifiable by its strength and permanent position along the equator. The situation is somewhat different for the distinction between the SEUC and the SECC since the meridional positions of their cores varies seasonally and overlap partly as visible in Fig 3.

The discussion of the water mass transport between the tropics and the subtropics focuses in the first place on the subsurface currents since the strongest surface equatorial current, the South Equatorial Current (SEC), is directed to the west. It appears above the EUC (Fig 2) and does not contribute to the tropical-subtropical coupling. Other important surface currents lie within the northern Benguela upwelling system and will be discussed later. Fig 3 shows the subsurface currents averaged from 50m to 200m depth. Figures of the surface currents and the currents averaged from 200m to 550m are supplied as supplementary material (S1 File).

The equatorial currents form a broad, structured subsurface flow band that is directed to the east or southeast and reaches the southwestern African coast at different latitudes. The strongest signal is related to the eastward directed EUC, located directly on the equator. SEUC and SECC are slower currents than the EUC. The maximum horizontal velocities in the release regions of the tropical tracers are 0.66 ms^−1^, 0.43 ms^−1^ and 0.26 ms^−1^ (EUC, SEUC and SECC, respectively; monthly climatological data). The water within this flow band experiences upwelling in October, which is replaced by downwelling along the SECC path in April.

The continuation of the EUC off the African coast is obvious. Most studies have focused on this “coastal” propagation path within the coastal waveguide to prove the tropical-subtropical coupling in the Southeast Atlantic [39]–[42]. However, less attention has been paid to the southward-bending arm of the EUC, the SEUC and SECC, which carries tropical SACW into the northern Benguela upwelling system on an “oceanic” pathway. The eastward flowing EUC is split off and displaced towards the south at the latest at 8°W. This results in a characteristic branch of the EUC tracer, which will be discussed later.

The main direction of the modelled SEUC is southeast, although its strength varies seasonally (Fig 3). A downstream poleward shift of the SEUC is also observed [103], in agreement with observations in the Pacific Ocean [104] and with theoretical considerations [105]. The analysis of Rossby wave phase velocity in the tropical Atlantic [24] suggests that direction and time variability of the SEUC are governed by Rossby waves radiating from the African coast.

The SECC enters the northern Benguela directly at about 16°S. This coincides well with direct current measurements ([44], their Fig 19). It is sometimes regarded as the northern limb of the Angola Gyre, which is considered as a geostrophic flow in the Southeast Atlantic [106–108]. A cyclonic gyre centred around approximately 11°E, 9°S is barely identifiable in our subsurface currents in April, but completely lacks in October (Fig 3).

**Fig 4:**
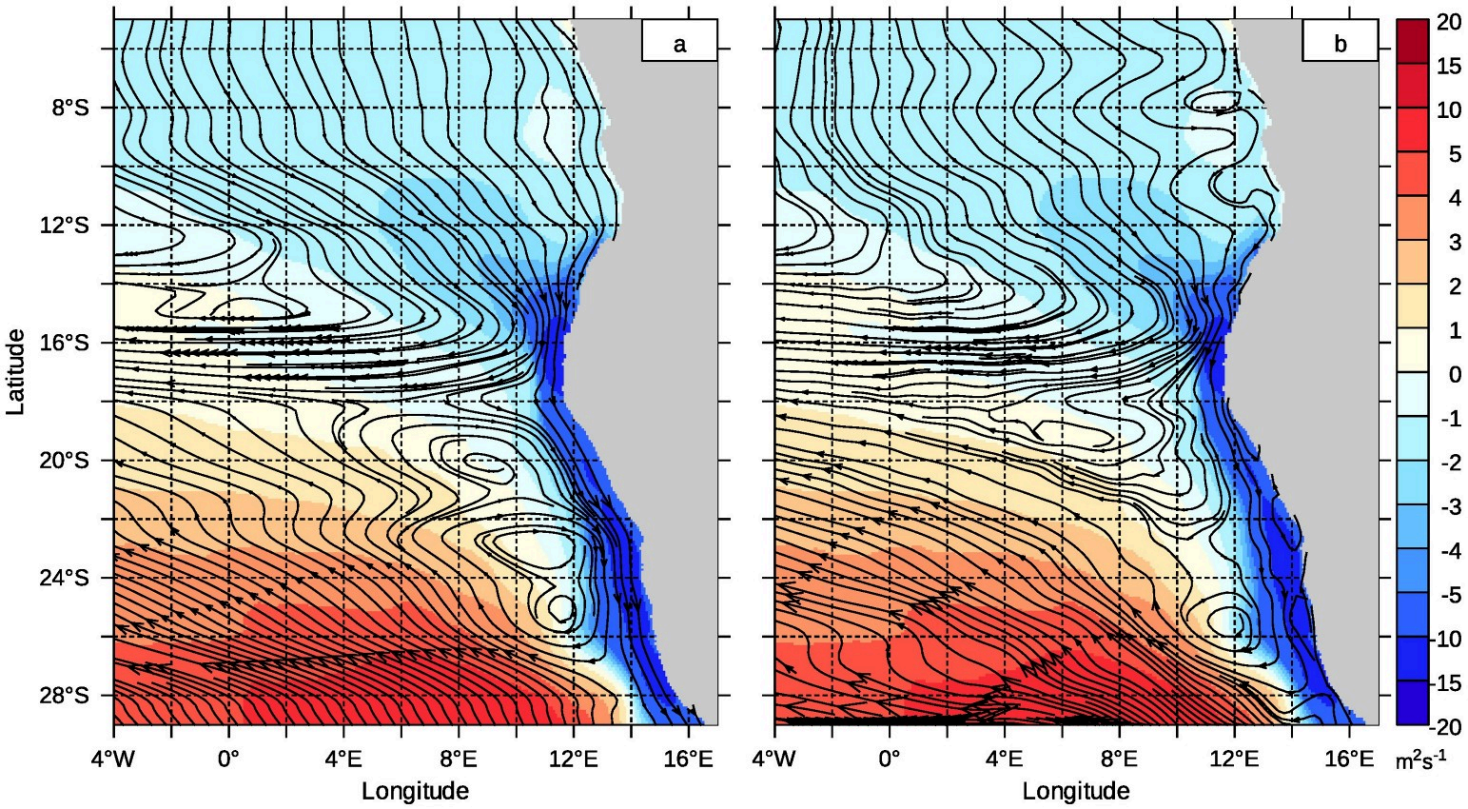
Sverdrup balanced averaged transport. Time averaged wind stress curl divided by density and Coriolis parameter (colour coded) and flow lines of the vertically integrated velocity. a) Flow lines of the Sverdrup balanced flow, b) Flow lines of the time averaged and depth integrated velocity (upper 500m) from the numerical circulation model.

Remarkably, the SECC flows against the prevailing wind. This suggests a Sverdrup balanced flow [46]. The strong negative wind stress curl off the Namibian coast and north-west off the Kunene Cell is able to balance a flow pattern with a significant poleward component. Fig 4a shows the time averaged wind stress and the related flow lines of the Sverdrup balanced barotropic transport derived from the stream function corresponding to Eq 1. Fig 4b shows the time averaged transport within the upper 500 m. Pattern and magnitude in both figures are very similar. North of 16°S, a broad south-westward flow is guided by the wind stress curl towards the African coast. The strong, localised patch with negative wind stress curl at 16°S is responsible for the sudden turn of the flow lines into a narrow westward directed stream band. The numerical results have a similar structure. Further south, the wind stress curl is positive and balances the flow of the north-eastern limb of the Subtropical Gyre. The negative wind stress curl stretching at the Namibian coast is important for the poleward water mass transport. The corresponding Sverdrup flow continues from the north into the Benguela and is also visible in the numerical model results. The magnitude of this flow is smaller than the Sverdrup flow, since an equatorward coastal jet often develops in the northern Benguela. This jet is not part of the Sverdrup theory and is missing in Fig 4a. The vertically integrated wind driven flow fluctuates around a mainly Sverdrup balanced average. The patch of negative wind stress curl in the Kunene Cell plays a key role for the interaction between the eastern tropical and subtropical Atlantic.

**Fig 5:**
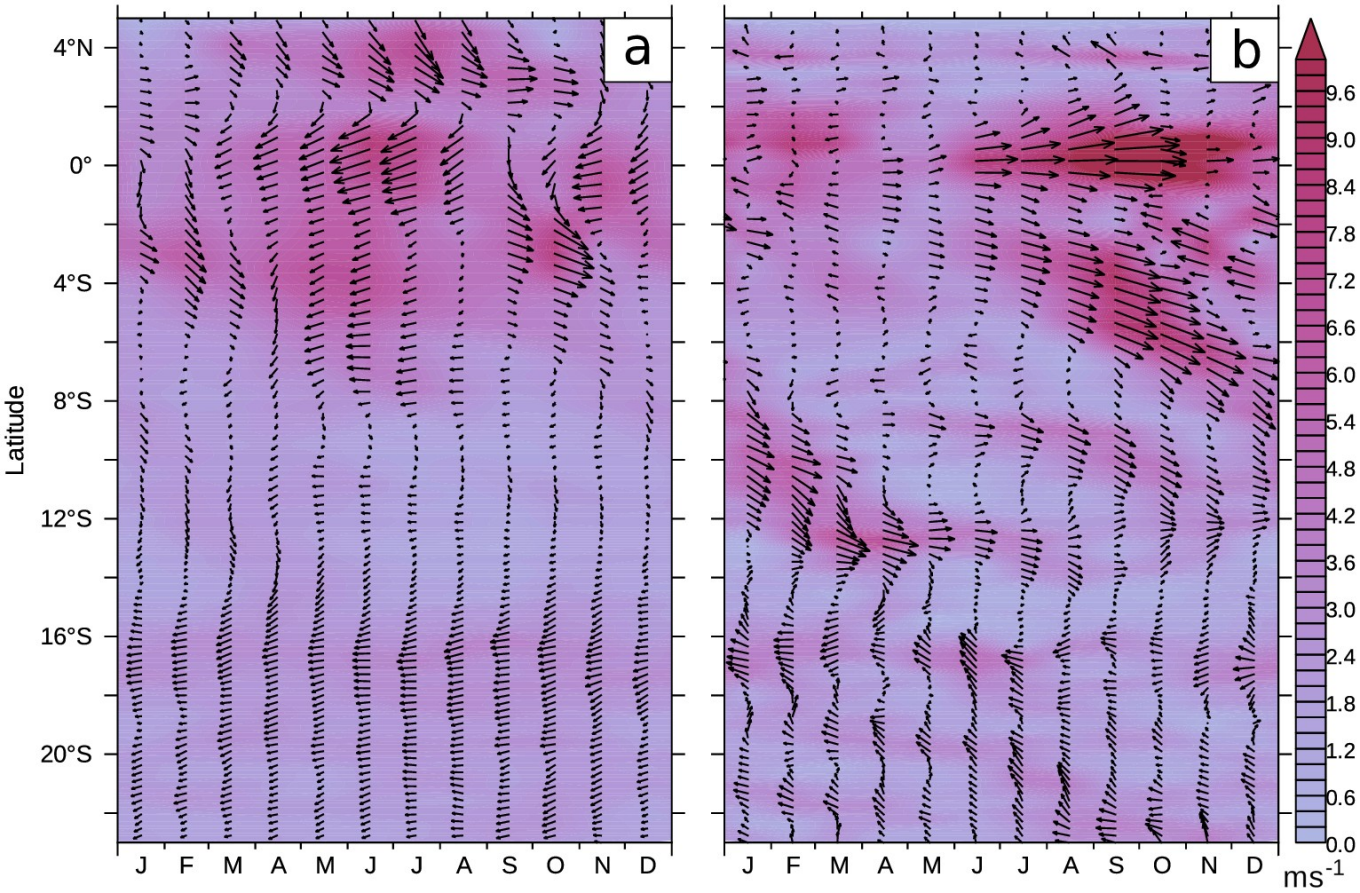
Hovmöller plot of horizontal currents at 5°E. The magnitude and direction of the vertically averaged horizontal currents at a meridional section at 5°E. a) Surface currents averaged from 0m to 50m b) Subsurface currents averaged from 50m to 200m. Monthly climatology from 2002 to 2016.

Nevertheless, a static view on the equatorial currents, especially on the position of the SEUC and SECC, does not seem consistent with the temporal variability of these currents (Fig 5). Remarkably, the meridional position of the flow band in southwestern direction shifts continuously poleward. The core of this flow is located at 2°S in July and can be found at approximately 13°S in March. We suspect that SEUC and SECC are only one expression of a regularly occurring current pattern produced by waves radiating from the Equator. The seasonal variability of the EUC with its highest velocities at 5°E in October is another striking feature in this figure. The surface SEC does not peak at 5°E at the same time, but peaks instead in June.

**Fig 6:**
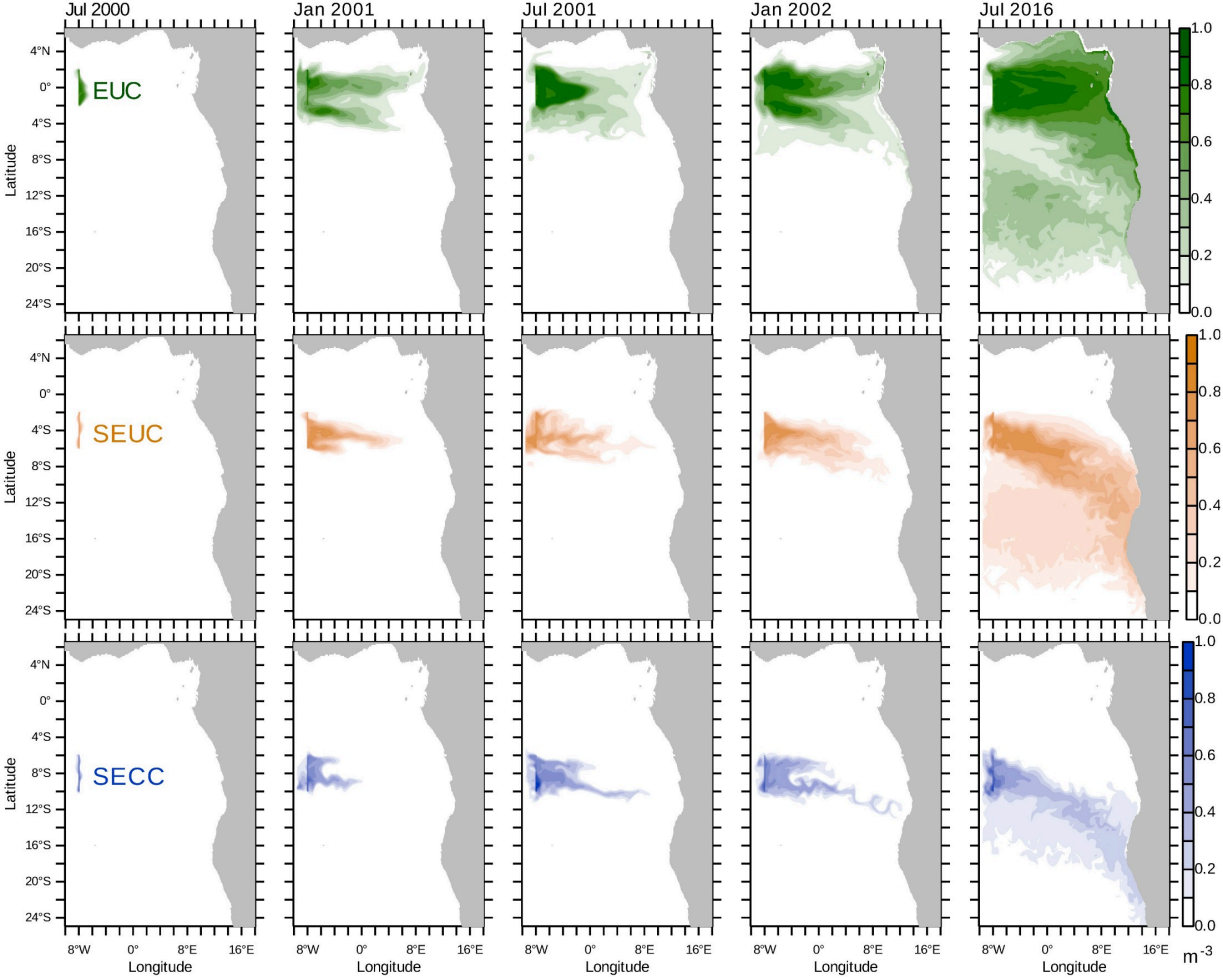
Tracer concentration isopycnicly averaged. The tracers are released at 8°W, 50m to 200m. Concentrations averaged within the isopycnal range given in Table 1 [m^−3^] are shown for July 2000 (a few days after tracer initialisation), January 2001, July 2001, January 2002 and July 2016. The tracers are released between 2°N to 2°S (EUC, upper panel), 6°S to 2°S (SEUC, middle panel) and 10°S to 6°S (SECC, lower panel).

The seasonal variability of SACW transport is further illustrated by tracer concentrations that result from long-lasting advection and diffusion processes (Fig 6). During austral winter, the EUC tracer concentration at the Equator has only one localised maximum. During austral winter, a second tracer maximum located at 4°W, 3°S forms each year and can be attributed to the subsurface southeastward flow (cf. Figs 3 and 5). The tracer originally released in the EUC reaches the African coast within this second propagation path at 12°S and then spreads along the coast.

The two EUC tracer concentration maxima visible in Fig 6 in January confirm a bifurcation of the EUC where one branch is bending southward and feeds into the SEUC and finally supplies water to the Benguela system. Consistent with the poleward bending of the EUC in our model, a thermocline EUC loss of 1.1 Sv along the Equator toward the south was described to feed upwelling between 2.5°S and 7.5°S [98].

Our results are also supported by Brandt et al. (2016, [102]). They demonstrated with a baroclinic modal decomposition both of model results and of velocity measurements at 23°W, 10°W and 0° that the annual cycle of the zonal velocity along the equator is dominated by the fourth baroclinic mode and the semi-annual cycle by the second mode. Both baroclinic modes show a southward bending of the EUC at about 10°E in both the realistic TRATL01 configuration (their Fig 6) and in three variations of a linear reduced-gravity model (their Figs 8–10).

After a longer period, a dichotomy establishes in the eastern tropical Atlantic (right panel of Fig 6). North of a line from 8°W, 2°S to 13°E, 13°S, more than 60% of the central water stems from the EUC. South of this line, water from the SEUC is replaced by water propagating with the SECC. Further south- and southwestward, the influence of these water masses is negligible. The patches of EUC and SEUC tracers in the west are formed by upwelled water that gets in contact with the atmosphere and looses its SACW characteristics. While drifting westward with the surface Ekman transport, the EUC and SEUC tracers are slowly entrained into the subsurface layer by deep mixing.

The waters from the EUC, SEUC and SECC merge in the coastal waveguide, but are not fully mixed there. The EUC water enters the coastal waveguide first and has the lowest potential density. It propagates poleward in the surface Angola Current. The SEUC tracer is released in water with a higher potential density and is usually found in the coastal waveguide below the EUC water (cf. Fig 2). The SECC tracer is released within water with even higher potential density and reaches the coast further south than the SEUC tracer, with the result that the SECC is not welled up in the Kunene Cell, but is transported poleward there. The maximum concentration of the SECC tracer is found below 100m depth.

**Fig 7:**
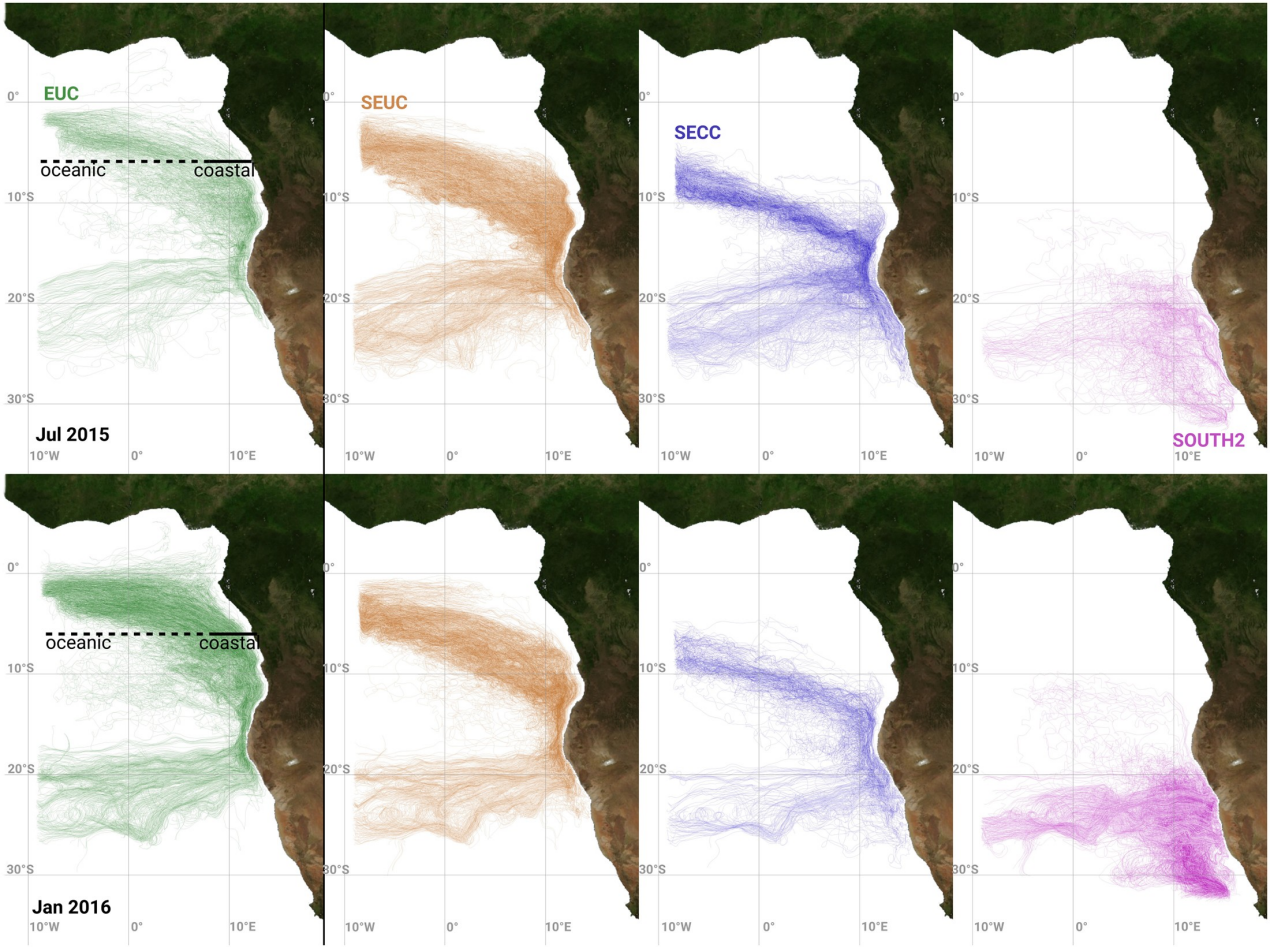
Upwelling trajectories. The lines visualise only trajectories of particles upwelled in the northern Benguela upwelling system. Upper panel: upwelling happens within the five days prior to July 13, 2015, lower panel: upwelling within five days prior January 14, 2016. For both time periods, the four panels show those trajectories of upwelling particles that traversed one of the tracer release regions EUC, SEUC, SECC and SOUTH2 (Table 1). Locations of “coastal” and “oceanic” EUC upwelling trajectories are indicated by the thick black lines.

Additional information on water mass spreading with the equatorial currents is obtained from trajectories of Lagrangian drifters. The farther south an equatorial particle starts, the farther south it reaches the Benguela upwelling area before it is upwelled (Fig 7). In addition, the area in which particles are upwelled changes seasonally. Between 9^th^ and 13^th^ of July 2015, the particles starting within the EUC are mainly upwelled in the coastal zone from 16°S to 19°S, particles starting within the SEUC from 16°S to 22°S and within the SECC from 17°S to 24°S. In January 2016, the EUC and SEUC particles are upwelled in the coastal zone from 18°S to 22°S. Particles transported with the SECC are upwelled south of 22°S. Particles originating from the southern model boundary (SOUTH2) are perennially not upwelled in larger quantities north of 20°S. The 20°S-latitude reveals to be the northern limit for the spreading of ESACW.

**Fig 8:**
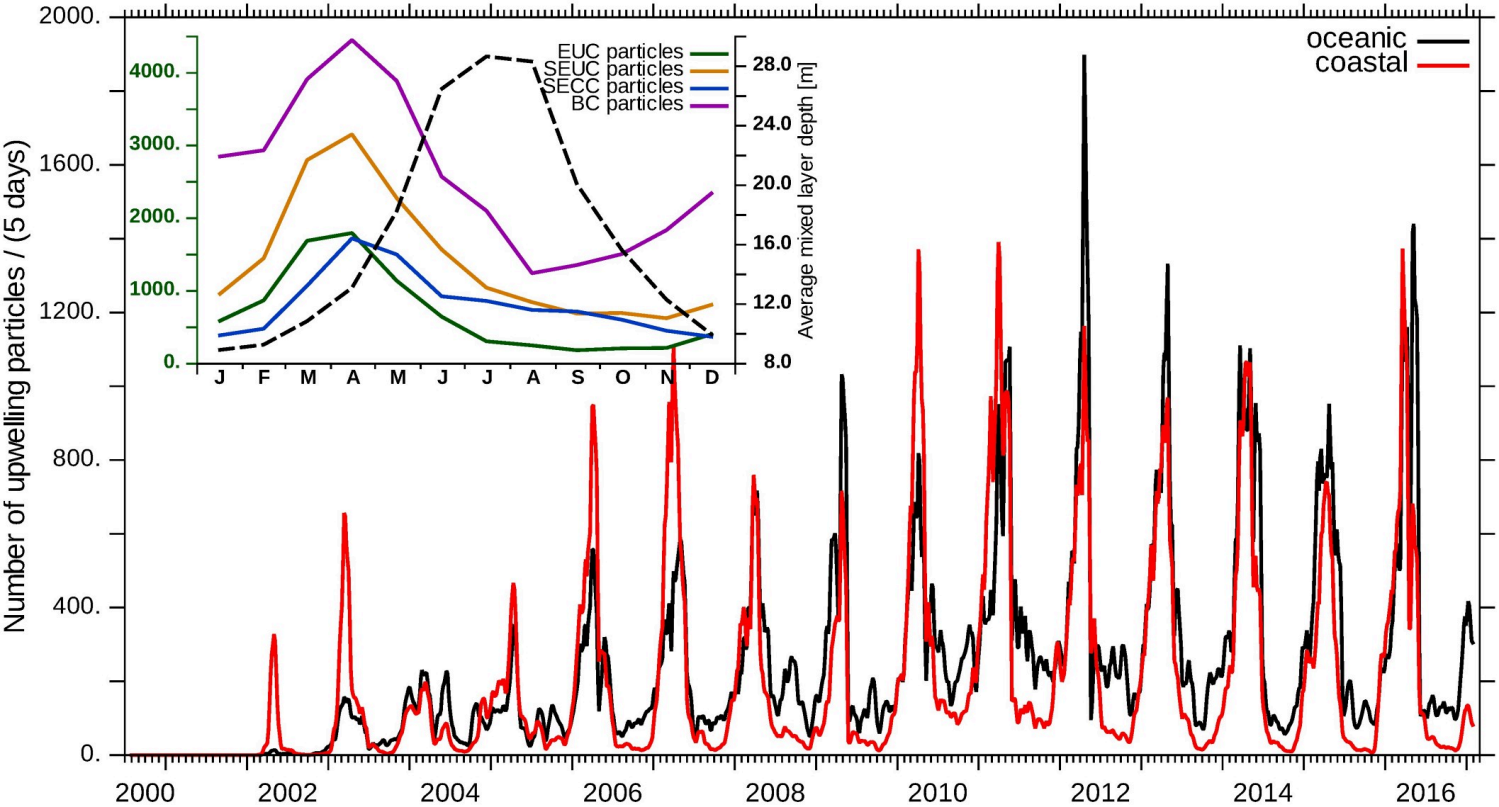
Coastal and oceanic propagation of upwelling particles. The number of upwelling particles per 5 d intersecting the release regions of the tracers show a strong seasonal variability. EUC upwelling trajectories were grouped as “oceanic” if they intersected a zonal section at 6°S between 10°W and 8°E, otherwise as “coastal” (cf. Fig 7). Data were smoothed with a running mean of 30d. Monthly climatologies of the number of EUC (green), SEUC (orange), SECC (blue) and BC (purple) upwelling particles per 5 d (2010 to 2016, axis on the left) and of the spatial average of the mixed layer depth in the northern Benguela (2002 to 2016, black dashed line, axis on the right) are shown in the upper left corner.

The Lagrangian trajectories help to distinguish upwelling particles propagating into the northern Benguela through the coastal waveguide from those propagating in the open ocean (Fig 8). On average, 60% of the EUC particles upwelling between January 1, 2003 and December 31, 2016 proceed in the open ocean before they are upwelled in the northern Benguela and the remaining EUC particles proceed in the coastal waveguide. In the months of maximum EUC upwelling particles (March to May), proportionally less “oceanic” particles are upwelled. The seasonal cycle of upwelling particles moving on both a “coastal” and an “oceanic” pathway into the Benguela is very similar. Until 2009, an equilibrium is established. However, upwelling particles from the “coastal” pathway prevail during two periods (2006/2007 and 2011/2012). The years 2011 and 2016 are exceptional since the “coastal” fraction is peaking two months prior to the “oceanic” fraction then. The number of upwelled particles reaches its maximum during April, when the mixed layer depth is increasing due to seasonal cooling and increasing wind mixing.

### Propagation of central water masses within the Benguela

Neglecting, in a first approximation, the spatial subdivision of the northern Benguela system in upwelling cells, a temporal variability of the upwelling strength is found (Fig 8). In March, April and May, the number of upwelling particles is highest, in August to November, it is lowest. Even without positive vertical current velocities, the number of upwelling particles can increase if the mixed layer depth increases. From May to July, the thickness of the mixed layer continues to increase while the number of upwelling particles already decreases. The contribution of SACW from the SEUC to upwelled water in the whole northern Benguela upwelling system seems to be quite important since more SEUC particles than EUC or SECC particles are upwelled year-round. Furthermore, the number of BC upwelling particles is highest compared to the tropical upwelling particles (EUC, SEUC, SECC), as its release region is much larger (cf. Table 1).

**Fig 9:**
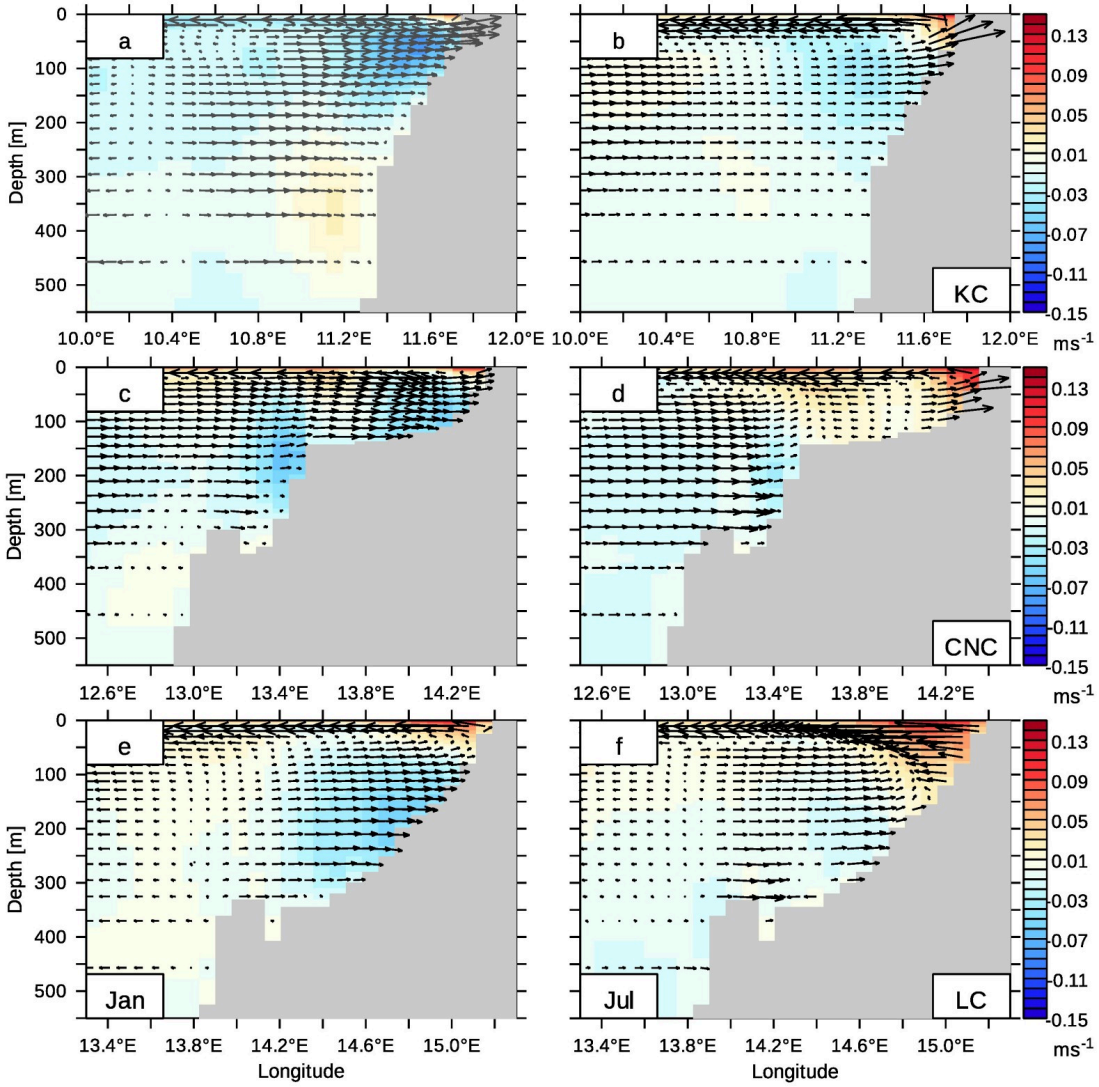
Currents in upwelling cells of the northern Benguela upwelling system. The currents [ms^−1^] at a zonal cross-section in the Kunene Cell (KC, 18°S; a and b), the Central Namibian Cell (CNC, 23°S; c and d) and the Lüderitz Cell (LC, 27°S; e and f). The vector arrows are composed of the zonal and vertical flow component. The vertical velocity component is enlarged for better visibility. Equatorward motion is shaded red. January (left) and July (right) from the monthly climatology from 2002 to 2016.

In the following, we turn to the spatial structure of upwelling in the northern Benguela. The currents in the upwelling cells determine how far south the tropical SACW can penetrate. Common to all upwelling cells is that an Ekman transport in the surface layer and an compensation current in the subsurface layer are induced by Southeast trade winds. Further offshore, the otherwise relatively uniform westward direction of the Ekman flow is disturbed by trails of Agulhas rings, which reach a few hundred meters deep. However, transport of SACW and ESACW is largely determined by the PUC and the coastal jet, respectively. The meridional (colour coded) and zonal (arrows) currents are shown in zonal sections through different upwelling cells, Fig 9. Within the Kunene Cell (KC), the shelf is relatively narrow and steep. The equatorward wind driven jet is located at the coast and flows with velocities of up to 11.9 cm s^−1^ in July and up to 9.4 cm s^−1^ in January. The PUC, which is crucial for poleward transport of tropical SACW, is located below this jet and has maximum velocities of 12.8 cm s^−1^. The surface Ekman flow carries a considerable amount of upwelled SACW from the EUC offshore (Fig 6). An onshore Ekman compensation flow establishes below the offshore currents at depths of 50m to approx. 300 m.

The Central Namibian Cell (CNC) has a wide double-shelf structure. The first shelf edge is located at a water depth of about 150 m. The adjacent outer shelf drops off relatively steeply to a undersea ridge rising approximately 50m above the sea floor. A second shelf edge follows at a water depth of 350m and separates the outer shelf from the open ocean. This double-shelf structure of the Central Namibian Cell (23°S) allows the evolution of two upwelling centres in July, when upwelling is strong. One upwelling centre is directly at the coast and can be described with a classical upwelling theory assuming the coast to be a solid wall. The other upwelling centre is found above the first shelf edge and can be attributed to “secondary” upwelling above the shelf edge [109]. In January, two centres of the PUC evolve: one on the shelf below the coastal jet and the other one above the first shelf edge. In July a coastal PUC is not visible, but masked by a stronger barotropic equatorward flow on the whole shelf.

The propagation of water originating from the boundary between SACW and AAIW was traced with a tracer released at 550m depth. T550 concentrations reach maximum surface values of 0.032 m^−3^ on the coast of the Lüderitz Cell. North of 25°S, in the northern Benguela upwelling area, the T550 concentrations do not exceed 0.016 m^−3^ during 16 model years. Hence, we find that the AAIW in the upwelled water occurs only in small quantities during the run time of our numerical model and hardly plays any role on the seasonal to annual time scale. This finding motivates the restriction of the analysis to the upper 550m of the water column. On larger timescales (several decades), we cannot exclude that AAIW comes to the surface in the Benguela. Since upwelling in the form of positive vertical velocities does not take place below 300m of water depth, it can be assumed that vertical mixing rather than upwelling brings the AAIW tracer to the surface in a prolonged model run.

Up to this point, we have shown the following aspects of upwelling in the northern Benguela. Firstly, upwelling, i.e. the upward directed motion of water, is variable over time. Neglecting the spatial subdivision of the system into upwelling cells, the composition of the upwelled water does not vary with time. Secondly, shelf topography (along with local winds) modifies the upwelling circulation known from analytical theory, and thus the strength of upwelling and transport of the central water masses within the system.

**Fig 10:**
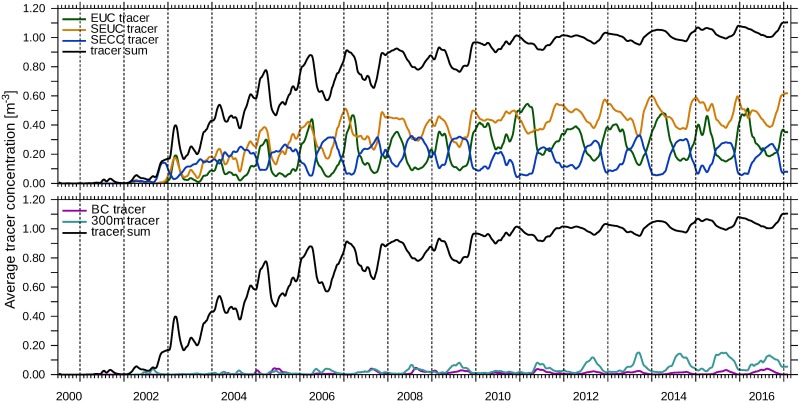
Central water in the Kunene Cell. The concentrations of tracers averaged over a cross-shore section from the coastline to the 200 m-isobath in the Kunene Cell at 18°S were computed with Eq 6. The tracers are released parallel to the western model boundary (EUC—green, SEUC—orange, SECC—blue) and parallel to the southern model boundary (BC—purple) between 50m to 200m. T300 is shown in turquoise. Additionally, the average concentrations of those five tracers are summed (black line in both panels). The corresponding release regions are given in Table 1.

Next, we will show how long it takes tropical SACW to reach the Benguela upwelling system. Furthermore, Fig 10 illustrates that the concentrations of the corresponding equatorial tracers are not in phase in the Kunene Cell. The EUC tracer arrives in the Kunene Cell after almost 1.5 years. The average tracer concentration then rises until 2011, i.e. for 12 model years, and finally reaches a kind of equilibrium state, around which it oscillates seasonally. Each year in December, the average concentration of the EUC tracer rises sharply, then slightly fluctuates around the level reached and drops in the months May to August.

The average concentration of the SEUC tracer varies with a seasonal cycle similar to the EUC tracer, with the long-term maximum not yet reached after 17 years of model run time. The SEUC-tracer concentration increases relatively quickly every November, reaches the seasonal maximum in December and falls until August or September.

The seasonal cycle of the average SECC concentration runs opposite to the cycle of the EUC and SEUC tracer. The concentration is minimal from December to March and becomes maximal for approximately 5 months in the second half of the year. The average concentration of T300 is in phase with the SECC tracer concentration in the Kunene Cell, but it does not reach its long-term maximum during the numerical experiment.

Presumably, this is because SACW originating from the EUC and SEUC enters the Benguela upwelling system as surface water. These waters are upwelled on their way to the Benguela (Fig 3) and are then carried offshore by Ekman transport in the Kunene Cell (Fig 6). The water from the SECC remains in its original depth on its way from the tropics to the Benguela, since it does not experience as much upwelling as EUC and SEUC waters. SACW from the SECC is then upwelled in the northern Benguela. In consequence, the average concentrations of the SECC tracer and T300 peak synchronously in the Kunene Cell.

**Fig 11:**
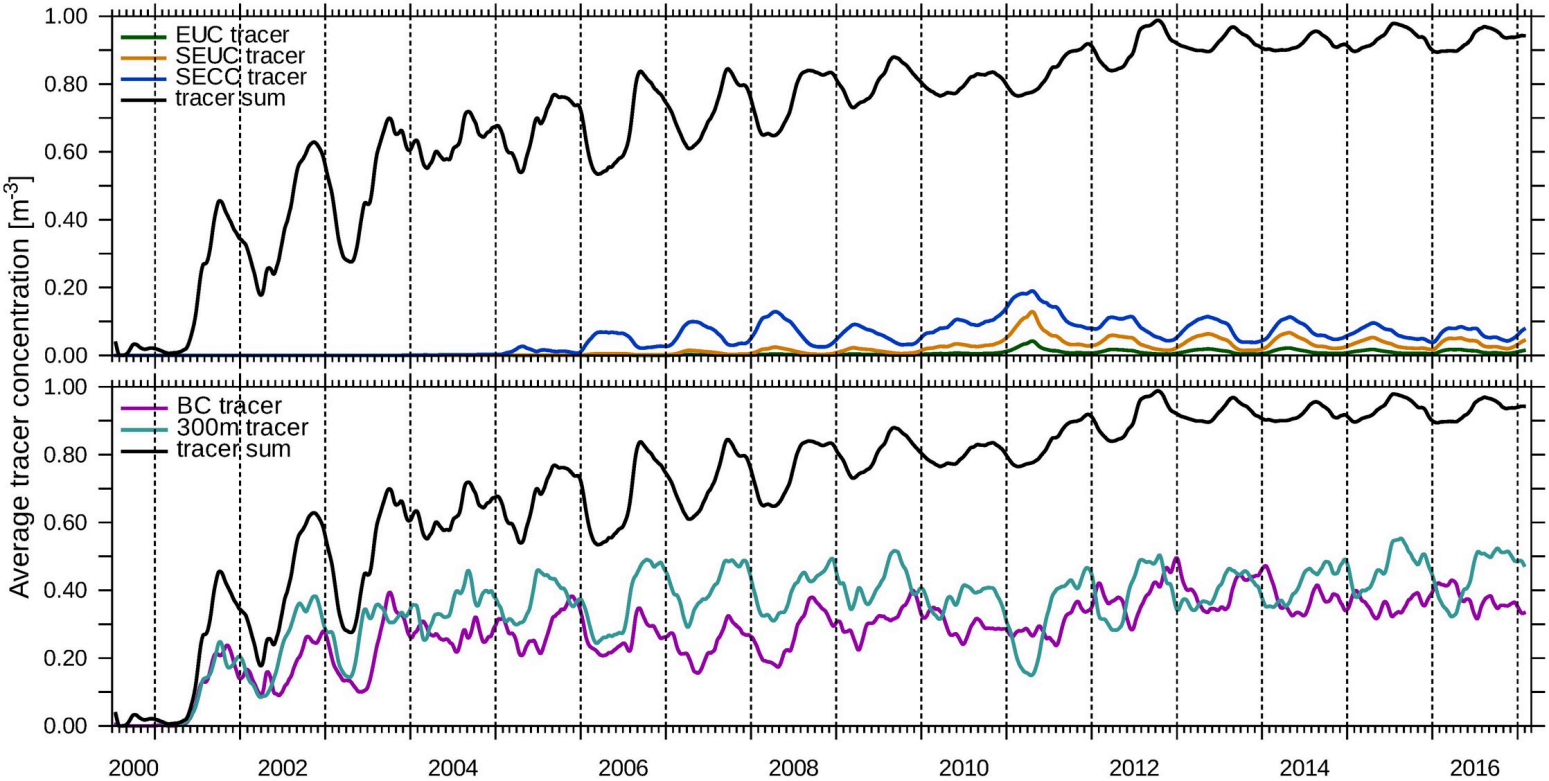
Central water in the Lüderitz Cell. The concentrations of tracers averaged over a cross-shore section from the coastline to the 200 m-isobath in the Lüderitz Cell at 27°S were computed with Eq 6. The tracers are released parallel to the western model boundary (EUC—green, SEUC—orange, SECC—blue) and parallel to the southern model boundary (BC—purple) between 50m to 200m. T300 is shown in turquoise. Additionally, the average concentrations of those five tracers are summed (black line in both panels). The corresponding release regions are given in Table 1.

The second water mass which feeds into the upwelled water is ESACW. The BC tracer is a proxy for subsurface ESACW that is carried with the Benguela Current into the northern Benguela upwelling system. Its average concentration in the Kunene Cell peaks in austral winter at the same time as the T300 concentration, but it is comparably low. A different picture emerges for the Lüderitz Cell in Fig 11. There, the average concentrations of the T300 tracer and the BC tracer are comparable in magnitude and exceed the values of the tropical tracers. In contrast, the BC tracer does not have such a pronounced seasonal cycle like in the Kunene Cell. Furthermore, the concentration of the BC tracer and the T300 tracer are less correlated than in the Kunene Cell. This is different for the average concentrations of the three tropical tracers that show a synchronous time course with seasonal maxima in April / May. The fact that all three tropical tracers are in phase there, indicates a common transport mechanism towards the Lüderitz Cell.

The results of the last paragraphs can be summarised as follows: On the Namibian shelf, a balance between subsurface SACW and ESACW with minor contributions of water from 300m depth is established, where one predominant water mass replaces another in the course of the year (Figs 10 and 11). In the northern Benguela upwelling system, tropical SACW encounters subtropical ESACW (Eastern SACW) [12]. Due to the seasonal variability of local wind forcing and current location, the position of the transition zone of the SACW and the ESACW changes over the course of the year. During austral summer, the shelf water in the northern Benguela mainly originates from the tropical Atlantic (Figs 10 and 11; [2], [11]). At this time, winds promoting upwelling are weaker ([24], their Fig 5) and poleward currents prevail in the upper 200m along the coast (Fig 9). Although the meridional transport in the northern Benguela is correlated to the local wind forcing, especially wind stress curl anomalies [45], the existence of the PUC is not purely bound to local upwelling-favourable winds, since Kelvin waves are also able to adjust a PUC poleward of the wind excitation area [110].

### Benguela Niño 2010/11

We identify the Benguela Niño event in 2011 on the basis of enhanced EUC tracer concentration in the Kunene Cell and the Lüderitz Cell (Figs 10 and 11). It already starts in September 2010 with an increased EUC tracer concentration and continues in the form of abnormally high concentrations even in the following austral winter. In the Kunene Cell, not only the seasonal minimum and maximum SEUC-tracer concentrations vary, but also the timing of the seasonal cycle is shifted. In this year, the SEUC concentration rises earlier (starting from August 2010), reaches a lower maximum value and decreases already from mid-November. Less water is transported into the Kunene Cell by the SECC. In spite of that, the T300 concentration shows a clear signal in the Kunene Cell, the winter maximum of 2010 is lower compared to other years. Our results suggest that the horizontal advection of larger quantities of equatorial warm water is not the only reason for the development of the 2010/11 Benguela Niño. Reduced upwelling is generally accompanied by a weakening of the Ekman transport and of the coastal jet in the surface layer. Therefore, the Kunene Cell becomes concurrently more permeable to surface water from the north and the flow on the shelf is directed poleward. In the Lüderitz Cell, the concentrations of all three tropical tracers reach their long-term maximum whereas the concentration of T300 is reduced to half of the normal seasonal minimum.

The weakening of coastal upwelling during Benguela Niño phases may result either from the excitation of equatorial Kelvin waves, which transport a downwelling signal to the Namibian coast, or from the weakening of the local wind field, which under normal conditions drives coastal upwelling. A description of comparable Kelvin wave dynamics in the Pacific during El Niño can be found in [113]. We consider the higher sea surface temperatures found in the northern Benguela upwelling system in 2010/2011 to be related to both increased transport of warm tropical SACW and reduced upwelling of cool water.

Currently, two processes that can trigger a Benguela Niño are discussed in the literature [114]: reduced trade winds over the Equator in January/February combined with a deepening of the thermocline along the Angolan coast [17, 20, 115, 116] or the influence of local wind stress [43]. To investigate the effect of wind forcing on the evolution of Benguela Niños would require case studies with a larger model domain covering the western equatorial Atlantic.

### Water mass composition in the mixed layer

**Fig 12:**
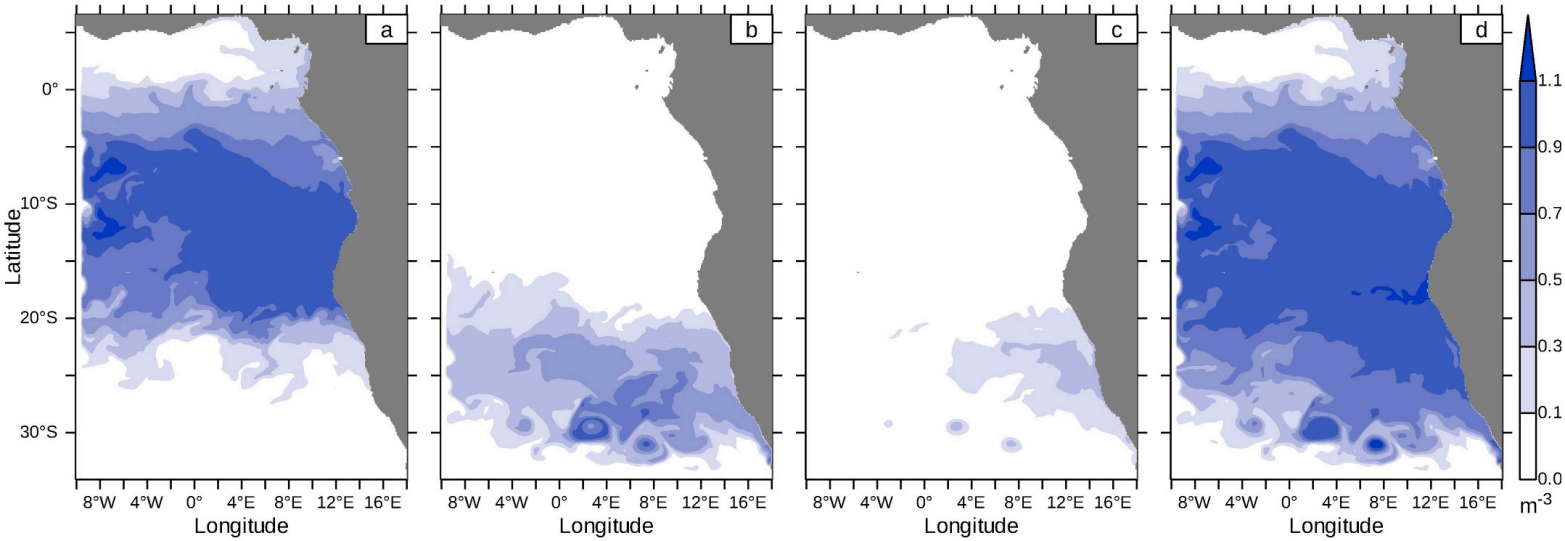
Vertical average concentration of tracers in the mixed layer. The average concentration of tracers from the western model boundary (a: sum over EUC, SEUC, SECC and WEST concentration), the southern model boundary (b: sum over concentrations from SOUTH 1 to SOUTH 4) and of T300 (c). The vertical average in the mixed layer of the sum over all nine tracer concentrations is shown in the right panel (d). Data were averaged from 11th to 15th of January, 2017.

In the following, we identify all remote sources of waters finally forming the mixed layer water with tracer experiments. Such is the case if the sum of all tracer concentrations is equal to unity. Fig 12a shows the sum of all tracer concentrations entering the model domain through the western model boundary averaged over the mixed layer. Analogously, Fig 12b and 12c show the vertically averaged sum of all passive tracers from the southern model boundary and the T300 tracer representing water upwelling from deeper layers.

The tropical tracers EUC, SEUC and SECC carry the tropical SACW in the subsurface layer until they are upwelled along the Angolan and Namibian coasts (Fig 12a) and dominate mixed layer water in the open ocean from 5°S to 20°S. Along the Equator, the mixed layer is composed of surface water (cf. Fig 3) and is too shallow to show traces of tropical water originating below 50m depth. Extratropical water from the western model boundary south of 10°S (tracer WEST) leaves the model domain with the South Equatorial Current (SEC), Fig 3.

The southern tracers representing the subsurface ESACW, Fig 12b, are transported by the Benguela Current into the model domain, but do not extend much further north than approximately 18°S, i.e. north of the Kunene Cell. Fig 12a and 12b show the transition zone of SACW and ESACW at about 20°S.

The tracer from 300m depth (Fig 12c) does not contribute significantly to mixed layer water, except in Agulhas rings (the eddy structures in the south) and in the Lüderitz Cell. The Agulhas rings propagate in a northwestern direction and have little effect on the Benguela upwelling system. In the northern Benguela, the combined effect of increased vertical mixing at sloping topography and coastal upwelling contributes to the upward motion of 300m-water. Since both the coastal jet and the upward directed velocities depend on the local wind field, it cannot clearly inferred from the T300 concentration in the Kunene Cell (Fig 10) whether the water is brought to the surface locally or whether the water is already upwelled in the Lüderitz Cell and is subsequently transported equatorward by the coastal jet.

Fig 12d demonstrates that the mixed layer water consists mainly of tropical SACW and subtropical ESACW in the Southeast Atlantic. All previously mentioned passive tracers were summed and the vertical mean in the mixed layer was calculated. The sum yields almost 1m^−3^. Values above 1m^−3^ are artefacts from the western model boundaries. Sometimes it happens that water flows successively through two tracer release regions. Except for the equatorial Atlantic and offshore areas off the southern Benguela, the vertical average of the summed tracer concentrations amounts to values between 0.9m^−3^ and 1.1m^−3^. Consequently, the water mass in the mixed layer between 5°S and 25°S is composed solely from subsurface SACW, ESACW and minor contributions of 300m water.

### Upper layer circulation in the Southeast Atlantic

**Fig 13:**
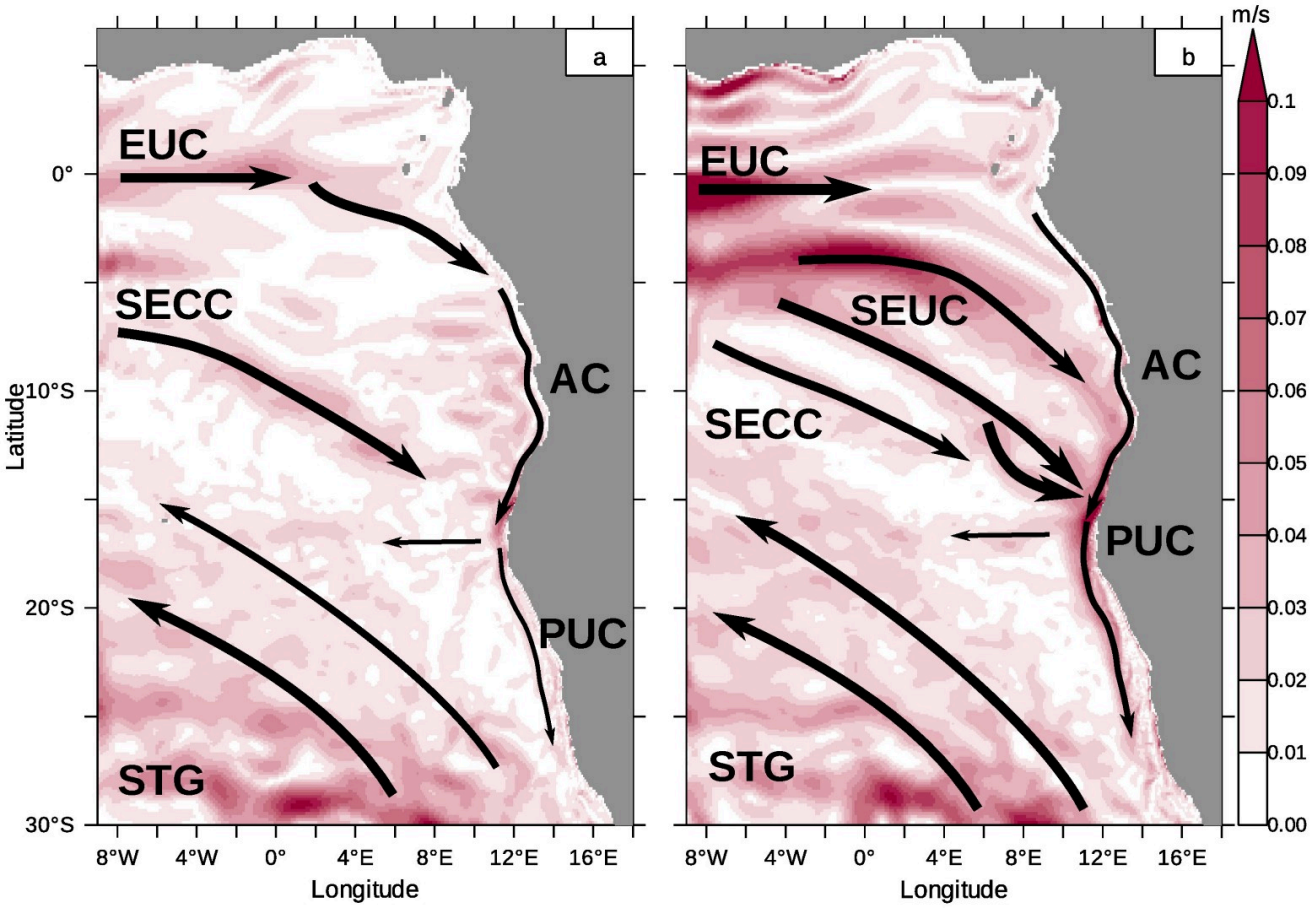
Schematic subsurface circulation. The magnitude of horizontal currents averaged over 50m to 200m is shaded. The black vectors represent main current systems. The current directions were derived from Fig 3. April (a) and October (b) of the monthly climatology from 2002 to 2016.

Finally, we present a revised scheme of subsurface circulation in the Southeast Atlantic. The scheme is based on the horizontal currents averaged from 50m to 200m for the months of April and October in Fig 13. The equatorial currents EUC, SEUC and SECC mediate the oceanic part of the tropical-subtropical coupling since they transport tropical SACW poleward. The EUC and its continuation as the Angola Current (AC) belong to the perennial features of the subsurface circulation. As mentioned above, it is difficult to associate the southeastward flow band in the tropical Atlantic with the SEUC or the SECC. Within the northern Benguela upwelling system, SACW is mainly transported with the PUC, ESACW flows equatorward with the coastal jet. The oceanic Benguela Current (BOC), to which the Benguela Current Large Marine Ecosystem owes its name, does not directly supply water to coastal upwelling areas off Namibia (Fig 7). Instead, the coastal BC (BCC) and an Ekman compensation current (Fig 9) are more likely to transport the subtropical ESACW to the coast. We interpret the surface BCC along the coast as a coastal jet, which is an integral part of upwelling circulation. In July, this coastal jet is so deep (Fig 9) that no PUC appears on the relatively shallow shelf south of approximately 23°S. In contrast, the coastal jet is weaker in January, the PUC can penetrate further south and carry tropical SACW accordingly. Transport flux budgets for the northern Benguela shelf confirm this observed seasonal variability of the meridional currents [111, 112]. The seasonal variability of the meridional extension of the PUC is related to wind stress curl [45]. The oceanic branch of the Benguela Current (BOC) is a salient feature in the subsurface and central layer all year and shows traces of passing Agulhas rings. It continues as South Equatorial Current (SEC), which together form the eastern limb of the Subtropical Gyre (STG, [21, 24]).

## Conclusion

Within this study, the advection pathways of central water in the Southeast Atlantic and their temporal variability were investigated with Eulerian passive tracers and Lagrangian trajectories from a regional hydrodynamic model. The main results are summarised as follows:

- There are two major transport paths for South Atlantic Central Water (SACW) originating from the tropical Atlantic to the Benguela upwelling system. Tropical SACW spreads with the Equatorial UnderCurrent (EUC), i.e. within the well-known Kelvin waveguide along the equator and its poleward continuation along the coast, and within the southern equatorial counter current system (South Equatorial UnderCurrent—SEUC and South Equatorial CounterCurrent—SECC). It bends southeastward between 4°W and 4°E and reaches the African shelf north of the Angola-Benguela Frontal Zone (ABFZ). The velocity and position of the equatorial currents show a strong seasonal variability.
- The poleward subsurface transport of SACW takes place against the prevailing wind direction and can be explained by a Sverdrup balance.
- Propagtion pathways of the central water masses were traced by Eulerian passive tracers and Lagrangian trajectories. Both methods provide similar results and illustrate the close link from the equatorial current system to the northern Benguela upwelling system. The feedback from the subtropical Atlantic to the tropical Atlantic could not be described, since the model domain does not cover the western Atlantic basin.
- In the northern Benguela upwelling system, the shelf water is composed exclusively of tropical SACW and subtropical ESACW (Eastern SACW). The upwelled water in the surface layer originates from these central water masses from depths of up to 300m.
- The seasonal variability of the composition of the upwelled water depends on latitude. The meriodional spreading of tropical SACW is limited to the south by the Lüderitz Cell, while subtropical ESACW is restricted to the north by the ABFZ. Consequently, the northern Benguela upwelling system is a transition zone of both central water masses with poleward decreasing SACW fraction.
- Inter-annual changes in the composition of upwelling water might be related to Benguela Niños. During the Benguela Niño of 2010/11, the transport of equatorial SACW was increased and the local upwelling was strongly reduced. The observed temperature anomaly is a result of increased poleward transport of warm water and by changes in the heat balance of the surface layer due to reduced upwelling of cold subsurface water.

Future work is necessary to investigate the consequences of climate change on the water mass distribution in the northern Benguela upwelling system. Nevertheless, the presented results have revealed the model’s potential to carry out case studies with modified wind forcing. It can be assumed that the predicted poleward shift of the subtropical wind systems may also cause a poleward shift in the central water mass distribution and, hence, alter oxygen and nutrient supply to the Benguela upwelling system.

## Supporting information

S1 Fig Comparison of modelled SST with Reynolds daily SST. The SST was averaged zonally over a coastal strip of 1° width. The black line shows the meridional position of the 20°C-isotherm from the Reynolds daily SST. The red line shows the 21°C-isotherm of the modelled SST. The blue line shows results from a model driven with ERA-interim winds.
(TIF)

S1 File Currents in the surface and the central layer. Horizontal currents averaged from 0m to 50m (surface layer, Fig 1) and from 200m to 550m (central layer, Fig 2) depth from a monthly climatology from 2002 to 2016. Colours represent the vertical velocity at 200m depth. Positive vertical velocity (red) is directed upward. (a): April, (b) October.
(PDF)
