# Supplementary figures and images for: The tropical-subtropical coupling in the Southeast Atlantic from the perspective of the northern Benguela upwelling system
Source: PLoS One. 2019 Jan 22;14(1):e0210083. doi: 10.1371/journal.pone.0210083 (PMC6342443; doi:10.1371/journal.pone.0210083)

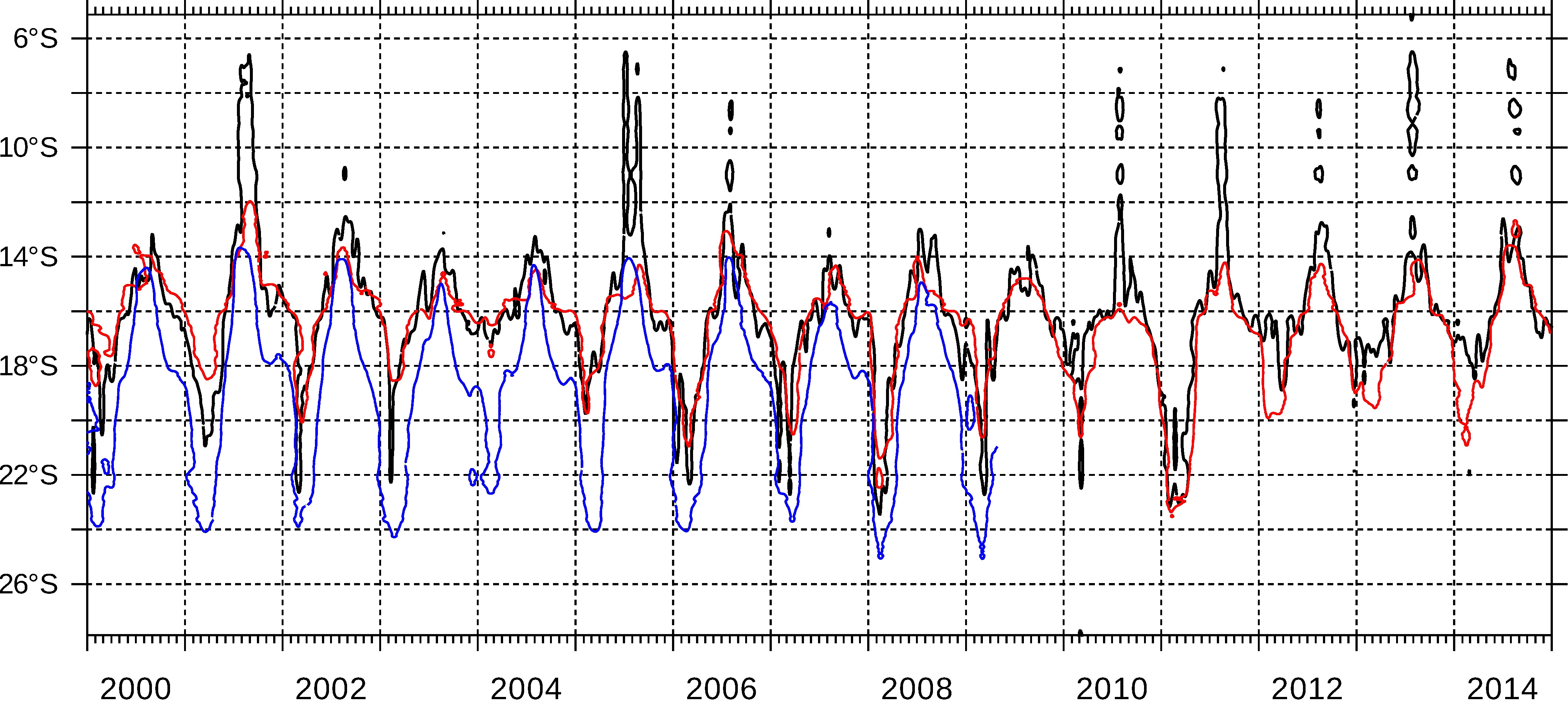

Supplement: S1 Fig — The SST was averaged zonally over a coastal strip of 1° width. The black line shows the meridional position of the 20°C-isotherm from the Reynolds daily SST. The red line shows the 21°C-isotherm of the modelled SST. The blue line shows results from a model driven with ERA-interim winds. (TIF) [file pone.0210083.s001.tif]
