# Supplementary material for: The tropical-subtropical coupling in the Southeast Atlantic from the perspective of the northern Benguela upwelling system
Source: PLoS One. 2019 Jan 22;14(1):e0210083. doi: 10.1371/journal.pone.0210083 (PMC6342443; doi:10.1371/journal.pone.0210083)
Supplement: S1 File — Horizontal currents averaged from 0m to 50m (surface layer, Fig 1) and from 200m to 550m (central layer, Fig 2) depth from a monthly climatology from 2002 to 2016. Colours represent the vertical velocity at 200m depth. Positive vertical velocity (red) is directed upward. (a): April, (b) October. (PDF) [file pone.0210083.s002.pdf]

Supporting information

Surface currents in the Southeast Atlantic

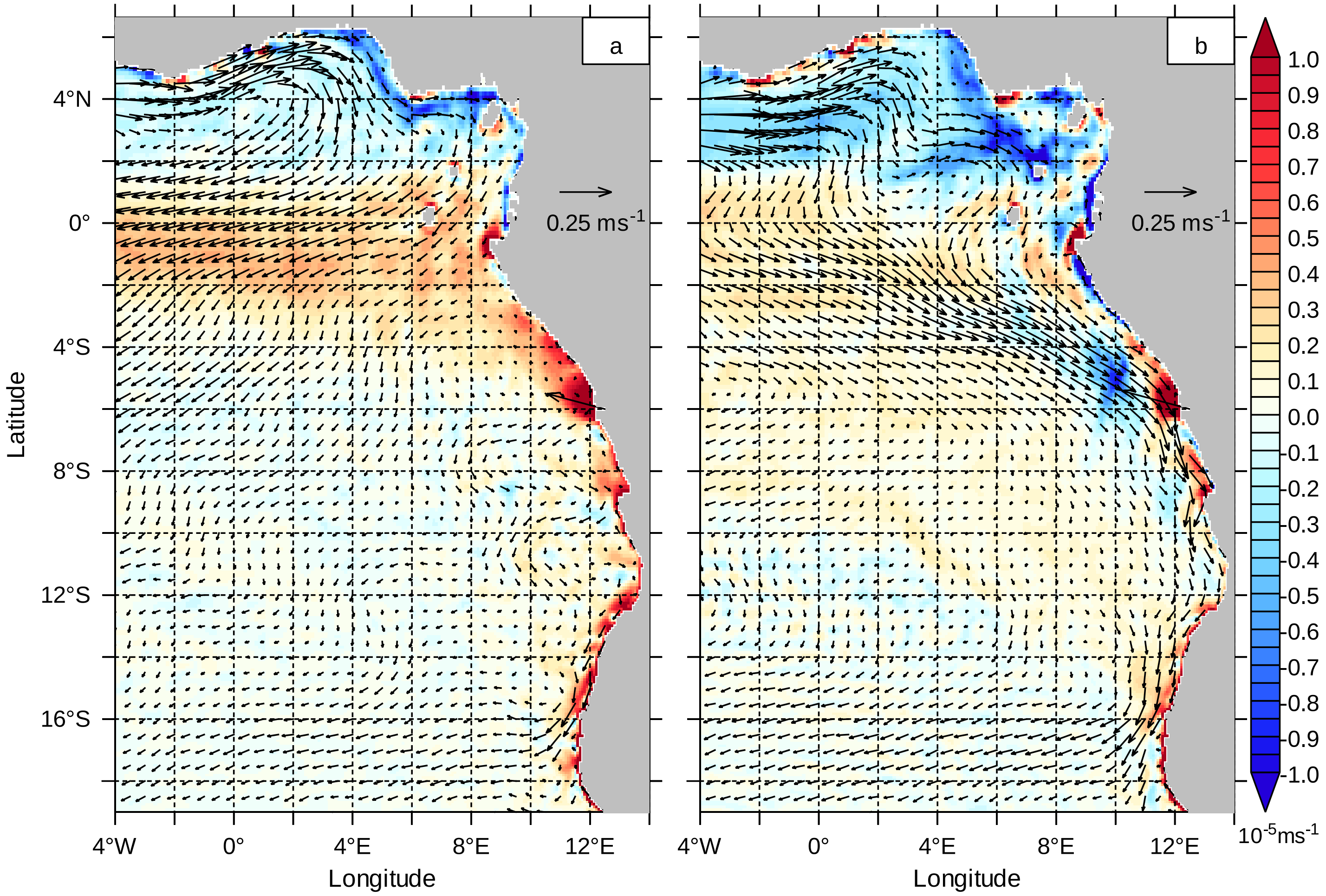

Fig 1. Currents in the surface layer Horizontal currents averaged from 0 m to 50 m depth from a monthly climatology from 2002 to 2016. Colours represent the vertical velocity at 10 m depth. Positive vertical velocity (red) is directed upward. (a): April, (b) October.

Central currents in the Southeast Atlantic 3

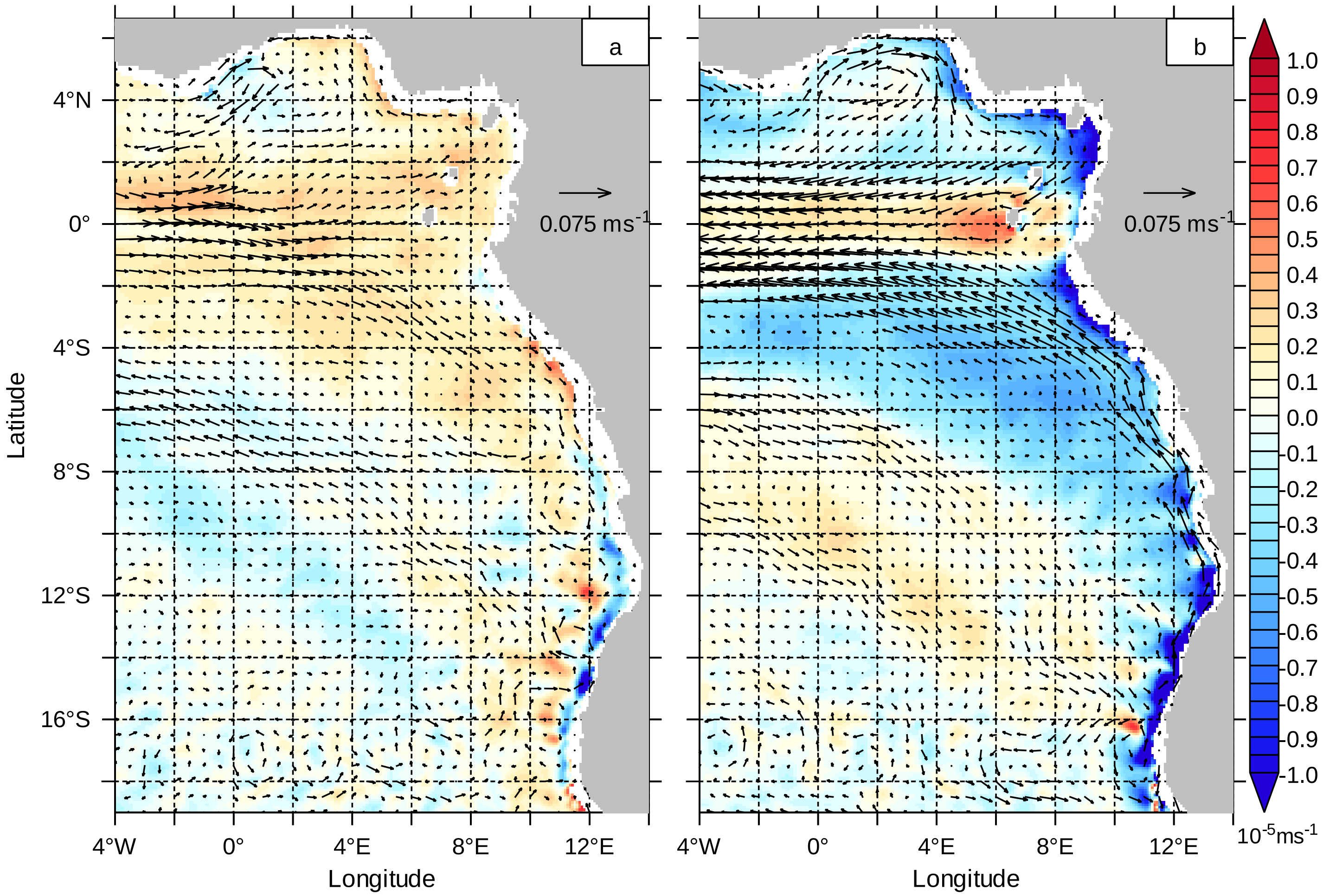

Fig 2. Currents in the central layer Horizontal currents averaged from 200 m to 550 m depth from a monthly climatology from 2002 to 2016. Colours represent the vertical velocity at 200 m depth. Positive vertical velocity (red) is directed upward. (a): April, (b) October.
